# Variations of stomata development in tea plant (*Camellia sinensis*) leaves in different light and temperature environments and genetic backgrounds

**DOI:** 10.1093/hr/uhac278

**Published:** 2022-12-09

**Authors:** Ping Li, Junming Lin, Mingzhi Zhu, Hao Zuo, Yihua Shen, Juan Li, Kunbo Wang, Penghui Li, Qian Tang, Zhonghua Liu, Jian Zhao

**Affiliations:** State Key Laboratory of Tea Plant Biology and Utilization, Anhui Agricultural University, Hefei 230036, China; Key Laboratory of Tea Science of Ministry of Education, College of Horticulture, Hunan Agricultural University, Changsha 410128, China; Key Laboratory of Tea Science of Ministry of Education, College of Horticulture, Hunan Agricultural University, Changsha 410128, China; State Key Laboratory of Tea Plant Biology and Utilization, Anhui Agricultural University, Hefei 230036, China; State Key Laboratory of Tea Plant Biology and Utilization, Anhui Agricultural University, Hefei 230036, China; Key Laboratory of Tea Science of Ministry of Education, College of Horticulture, Hunan Agricultural University, Changsha 410128, China; Key Laboratory of Tea Science of Ministry of Education, College of Horticulture, Hunan Agricultural University, Changsha 410128, China; State Key Laboratory of Tea Plant Biology and Utilization, Anhui Agricultural University, Hefei 230036, China; College of Horticulture, Sichuan Agricultural University, Chengdu 611130, China; Key Laboratory of Tea Science of Ministry of Education, College of Horticulture, Hunan Agricultural University, Changsha 410128, China; State Key Laboratory of Tea Plant Biology and Utilization, Anhui Agricultural University, Hefei 230036, China; Key Laboratory of Tea Science of Ministry of Education, College of Horticulture, Hunan Agricultural University, Changsha 410128, China

## Abstract

Stomata perform important functions in plant photosynthesis, respiration, gas exchange, and interactions with environments. However, tea plant stomata development and functions are not known. Here, we show morphological changes during stomata development and genetic dissection of stomata lineage genes regulating stomata formation in tea developing leaves. Different tea plant cultivars displayed clear variations in the stomata development rate, density and size, which are closely related to their tolerance against dehydration capabilities. Whole sets of stomata lineage genes were identified to display predicted functions in regulating stomatal development and formation. The stomata development and lineage genes were tightly regulated by light intensities and high or low temperature stresses, which affected stomata density and function. Furthermore, lower stomatal density and larger size were observed in triploid tea varieties as compared to those in diploid plant. Key stomata lineage genes such as *CsSPCH*s, *CsSCRM,* and *CsFAMA* showed much lower expression levels, whereas negative regulators *CsEPF1* and *CsYODA*s had higher expression levels in triploid than in diploid tea varieties. Our study provides new insight into tea plant stomatal morphological development and the genetic regulatory mechanisms on stomata development under abiotic stresses and genetic backgrounds. The study lays a foundation for future exploring of the genetic improvement of water use efficiency in tea plants for living up to the challenge of global climate change.

## Introduction

Stomata are the small pores surrounded by a pair of guard cells (GCs) on the leaf surface, and they allow CO_2_, O_2_, H_2_O, as well as other volatiles in and out of plants [1, 2]. Besides being for photosynthesis and gas exchanges, stomata are also the enter portal for bacterial pathogens and regarded as a battlefield in the plant innate immune system [1, 2, 3, 4]. Stomata exhibit diverse patterning in stomata density and size, guard cell shape, and subsidiary cells among different plants [2, 3, 4]. Stomata can be developed either on both leaf surfaces or a singular surface, depending on plant species for adapting to specific environments [5, 6, 7, 8].

Not only could stomata development be affected by light and temperature changes, but stomata pores change in response to other environmental factors, such as humidity and CO_2_ concentrations, in order to maximize photosynthesis efficiency but minimize water loss [6, 7, 8, 9, 10]. For instance, the limited water under dehydration or drought conditions, the reduced hydraulic conductivity, and increased abscisic acid (ABA) signals can induce guard cell turgor pressure decreases, and result in the reducedstomatal aperture or stomata close [9, 10]. The stomata movement responding to the environmental and hormonal cues plays a key role in controlling transpiration rate and water use efficiency, CO_2_ uptake for photosynthesis, as well as nutrient uptake [1, 9, 10]. Besides the stomata movement as a major way for the plant to adapt to water conditions, leaf stomata density, size and aperture change in response to other environmental factors, suchas low and high temperature, light or shade conditions, as wellas genetic factors [11, 12]. Plants usually modify their stomata development to adapt and survive the changing environmental stresses. This plasticity of stomatal development also enables plants to modulate water use efficiency and photosynthesis byregulating stomatal density, size, aperture, or movement [13].

Originated from meristematic protodermal cells (PCs) undergoing a series of asymmetric and symmetric divisions, stomataldevelopment is a tightly regulated process to pattern the leaf epidermis [1, 2, 4, 7, 14]. In Arabidopsis, the stomatal cell lineagebegins with asymmetric division of a young epidermal cell called a meristemoid mother cell (MMC), which can create a meristemoid cell (M) and a stomatal lineage ground cell (SLGC). The MC is a stomatal precursor that undergoes three asymmetric divisions before differentiating into guard mother cells (GMCs). A GMC further undergoes another single symmetric division to become a pair of GCs forming stomata. In addition, SLGCs can differentiate into pavement cells or divide into MMCs, which are termed as secondary MCs through asymmetric divisions to form GCs and orientation to prevent the formation of adjacent stomata [14]. A transcriptional regulatory network and few signal peptide-receptor interactions, feedback and feed-forward regulatory loops have been established to tightly regulate the stomata lineage, among which the transcriptional regulatory network is composed of positive bHLH and MYB regulators and negative signaling components that regulate cell fate and cell division patterns in the Arabidopsis stomatal lineage [7,15–18].

Generally, stomata development involves the secretary signal peptides Epidermal Patterning Factor 1 (EPF1)/EPF2, LRR receptor components Too Many Mouths (TMM) and Somatic Embryogenesis Receptor Kinases ERECTA (ER) or ERECTA-LIKE 1 (ERL1) and ERL2 that form receptor complexes, a serine protease Stomatal Density and Distribution 1 (SDD1), a mitogen-activated protein (MAP) kinase cascade that can phosphorylate and destabilize the basic helix–loop–helix (bHLH) transcription factor (TF) SPEECHLESS (SPCH), and then other bHLH TFs, such as MUTE and FAMA. EPF1 and EPF2 can activate the MAP kinase MAPK6 and decrease the SPCH level, whereas STOMAGEN (AtEPFL9) is able to increase SPCH level [18, 19]. EPF2 is produced in *SPCH*-expressing MMCs and regulates the number of cells that enter and remain in the stomatal lineage. EPF1 is produced in the late-stage MCs, GMCs and young GCs [8]. The Breaking of Asymmetry in the Stomatal Lineage (BASL) polarization regulates nuclear MAPK6 signaling and polarity-mediated differential suppression of SPCH [20, 21]. The stomatal lineage is sequentially regulated by bHLH and MYBTFs, SPCH, MUTE, SCREAM (SCRM), FAMA, and Four Lips (FLP)/MYB88 [8]. SPCH regulates the M phase of stomatal lineage; MUTE regulates the transition from MC to GMC, regardless of organ identity, by regulating *SOL1* and *SOL2*, cell cycle-related genes such as cyclins A and D (*CYCA* and *CYCD*) and Cyclin-Dependent Kinase A and B (*CDKA* and *CDKB*) [20].

The evergreen perennial tea plant (*Camellia sinensis*) is widely grown in more than 60 countries and areas for producing the most consumed popular non-alcohol beverage: tea [22]. The tea production in major tea-producer countries such as China, India, Sri Lanka, and Kenya suffered increasingly from extreme weather conditions due to global climate changes. Particularly in recent years, huge losses have been reported on global tea production due to severe weather conditions, such as spring freeze, drought stress, and extremely high or low temperatures. Understanding how tea plants adapt to these extreme weather conditions is highly desirable. However, few reports are seen on stomata in tea plants that can largely determine the drought tolerance and water use efficiency of tea plants. This study attempts to investigate the stomatal development in developing leaves of tea plants with cell biological approaches, stomata lineage genes involved in stomatal development, and developmental consequences of stomata under changing light intensity and temperatures or drought stress conditions. The study is expected to provide the first comprehensive insight into tea leaf stomata development in response to light, temperature, and drought stresses. The results could pave the road towards further understanding of tea plants in response to environmental stresses for better drought resistance and water use efficiency.

## Results

### The developmental state of stomata in tea plant developing leaves

**Figure 1:**
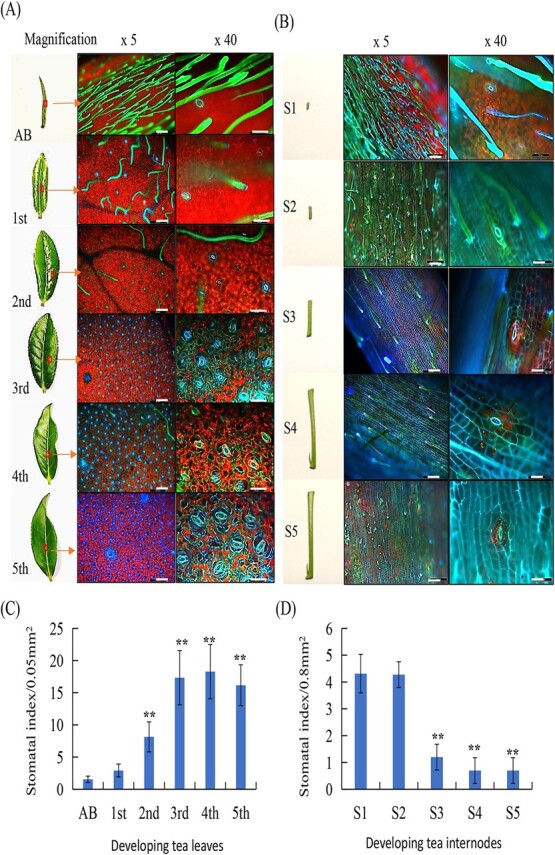
Morphological changes of developing stomata in tea plant leaves and stems. The apical bud (AB), the first, second, third, fourth, and fifth leaf as well as the corresponding internode (stem) of Shuchazao plants grown under regular conditions were used to observe under a fluorescence microscope. **A** The apical bud (AB), first leaf (first), second leaf (second), third leaf (third), fourth leaf (fourth), and the fifth leaf (fifth), and trichomes or stomata on it recorded under a fluorescence microscope with different magnifications. Scale bar = 500 μm; Scale bar = 200 μm (×5); Scale bar = 50 μm (×40). **B** The internode between AB and the first leaf (the first stem, in short as S1), and other corresponding internodes to the second, third, fourth, fifth stem, and trichomes or stomata on it recorded under a fluorescence microscope with different magnifications. Scale bar = 50 μm (×40), or Scale bar = 200 μm (×5). **C** and **D** Changes in stomata density in developing leaves and stems. Mature stomata were examined and counted for calculating the stomatal index under a microscope. At least three different areas in the middle leaf region of a leaf were imaged. Data were presented as means of 18 leaves from six individual tea plants (means ± SD, *n* = 18).

Firstly, we examined the stomatal morphology on the epidermis of tea plant leaf and stem, using ‘Shuchazao’ (SCZ) variety as materials. Stomata on the apical bud, the first, second, third, fourth, and fifth leaf were examined under a fluorescence microscope so as to clearly observe the number, shape, and development status of stomata. These images displayed clear morphological variations as tea plant leaves undergo development at six different stages. On apical buds, the leaf surface was covered by a lot of densely spaced trichomes, and only very few stomata were observed (Fig. 1A). On the first leaf surface, the number of stomata increased significantly. Meanwhile, many potential MMC were observed, indicating the stomatal initiation and starting to form young stomatal cells (Fig. 1A). On the epidermis of second leaf, stomata number further increased, and the sizes of stomata changed differently. On the third leaf, the number of stomata jumped to a much higher density than on the second leaf. Both young and mature guard cells displayed clearly different sizes (Fig. 1A, yellow arrow). On the fourth leaf, the density of stomata seemed slightly decreased as compared with that in third leaf, but the sizes of most stomata on fourth leaf were close to the same, indicating that most of these stomata were the matured ones, and leaf area expansion may cause the stomatal density decrease (Fig. 1A). On the fifth leaf, stomatal morphology and density were not changed compared with these on the fourth leaf. The stomata on these six representative leaves should represent the stomata morphological changes at six different developmental stages. Both adaxial and abaxial sides of tea plant leaves developed stomata, but the adaxial side had much less than the abaxial side of the leaf (Fig. 1A).

We further examined the stomata on the epidermis of different stems. The stem 1, referring to the first internode between the apical bud and the first leaf, was covered with many trichomes and had only a few stomata (Fig. 1B). On stem 2’s surface, the stomata number increased compared with stem 1 (Fig. 1B). The stomata on Stem 3, 4 and 5 became smaller and rare, only one to two stomata could be observed under a ten-fold lens (Fig. 1B). The overall development position of the stomata on the stem was on the top part beside the long stem epidermal cells, and most of the guard cells on the stem coincided with the cell elongation and growth direction of the tea tree. Thus, most stomata were on the leaf in tea plants. The number counts of stomata in apical buds and other developing leaves (Fig. 1C) showed that stomata number increased over leaf development and reached the maximal density at the third and fourth leaf, whereas it decreased over stem development. (Fig. 1D).

### Microscope observation of cell divisions toward stomata formation in tea leaf and stem

**Figure 2:**
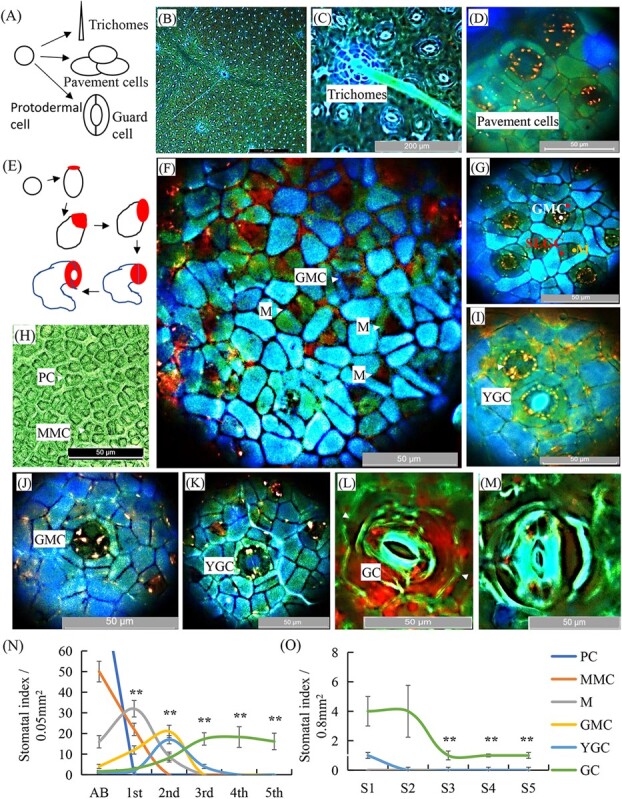
Microscopic observations of epidermal cell divisions and stomatal differentiation in developing tea plant leaves. **A** Schematic of leaf protodermal cell differentiation into either trichome, pavement, or guard cells. **B** Overview of leaf epidermal. Bright dots are stomata. Bar = 500 μm. **C** A unicellular trichome, pavement cells, and stomata. Bar = 200 μm. **D** Zoom-in views of several pairs of stomatal GCs. Bar = 50 μm. **E** Schematic of the initial asymmetric divisions followed by a symmetric division to form a pair of GCs. **F** Image of asymmetric divisions in forming stomata in tea apical bud epidermis. Abbreviations: GC, guard cell; GMC, guard mother cell; M, meristemoid cell; MMC, meristemoid mother cell; PC, protodermal cell; SLGC, stomatal-lineage ground cell. **G** Images of GMCs and stomatal lineage ground cells (SLGCs). **H** Images of PCs. Bar = 50 μm. **I** Initial guard cell and young GC. Bar = 50 μm. **J** Image of a GMC. Bar = 50 μm. **K** Image of a YGC. Bar = 50 μm. **L** Image of a young GC. Bar = 50 μm. **M** Image of a mature GC. Bar = 50 μm. **N** Changes in number of different cell types during leaf stomatal lineage formation. **O** Changes in number of different cell types during stem stomatal lineage formation. Different cell types and stomatal index were analysed in tea plants from the first leaf/stem to the fifth leaf/stem. Guard mother cell and SLGCs are pseudocolored in red and blue, respectively.

The epidermis of a plant developing leaf usually consists of PCs, which can differentiate into three main types of cells in Arabidopsis: trichome, pavement cell, and stomatal guard cell [8, 15]. In tea plant leaves, we also observed these three different types of cells on the epidermis, unicellular non-branching trichomes, guard cells, and pavement cells on the basis of trichome and around the guard cells (Fig. 2A–D; Fig. S1, see online supplementary material). A specialized epidermal lineage undergoes a series of cell divisions and successive cell-state transitions, which can be morphologically distinct. We tried to examine each transitional state in stomata formation. The stomata cell lineage begins with the physically asymmetric divisions of a young epidermal cell, MMC, to create a small triangular MC and a larger sister cell, SLGC (Fig. 2G). We observed many large MMCs in round-shape on apical buds. On the first leaf epidermis, the MCs have a transient stem cell-like property and experienced several rounds of divisions [8] (Fig. 2G). We were able to observe these type of cells in apical buds (Fig. 2G). Through several divisions, an MC and SLGC are produced (Fig. 2G). The differentiation of the round GCs and their patterned distribution in the epidermis are through asymmetric cell divisions (Fig. 2F and I). As a stomatal precursor, MCs complete three self-renewing asymmetric divisions before differentiating into GMCs. A GMC undergoes a single symmetric division to become the paired GCs of a stomata (Fig. 2G). SLGC can differentiate into pavement cells or may also become MMCs to initiate asymmetric interval division to produce secondary MCs, which are always located at the distal end of existing stomata or precursors (Fig. 2G). Eventually, the meristem is transformed into a GMC with obvious changes in cell shape and division ability (Fig. 2F). GMC divides symmetrically to produce two cells that could be observed on second and third leaves (Fig. 2I and N); and then undergoes the final cell state transition at the same time to form terminally differentiated young GCs, which could be observed on third leaves (Fig. 2L and N). Further, they develop into mature and more comprehensive mature guard cells, which were observed mostly on fourth and fifth leaves (Fig. 2I–M). Counts of different types of cells in stomatal lineage in apical buds and young leaves at various developmental stages showed the trends described above (Fig. 2N and O).

### Identification and expression patterns of stomatal lineage genes in tea plants

**Figure 3:**
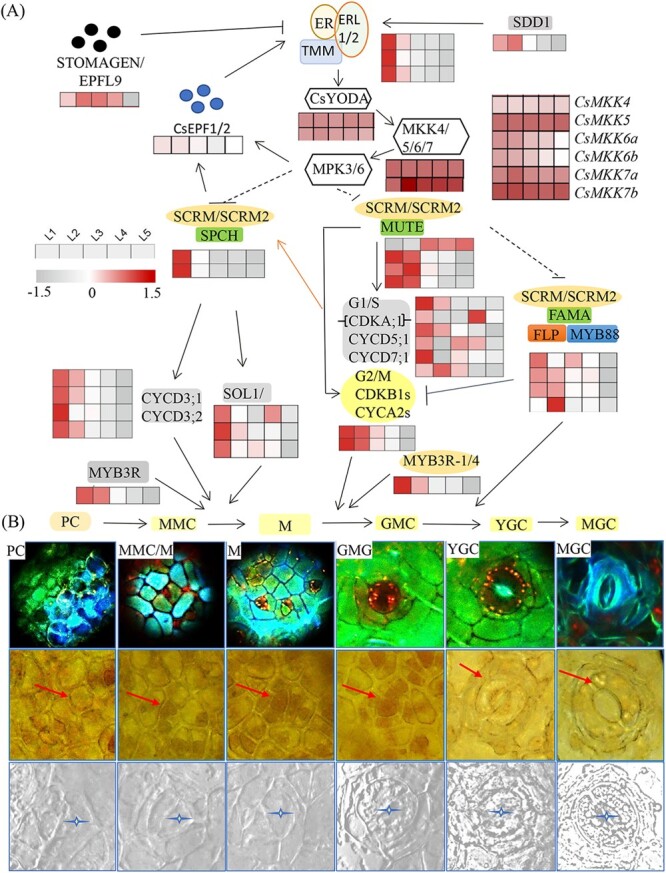
Schematic of signaling and regulatory networks initiating and regulating stomatal development in tea plants. **A** Conserved signaling components and regulatory factors initiating and controlling the stomatal development and their coding gene expression patterns in the developing leaf of tea plants. Tea plant has whole sets of stomatal lineage genes as counterparts of Arabidopsis homologs. The expression patterns of these genes in developing leaves were listed in heatmaps. **B** The regulatory targets of these genes in stomatal development processes are shown in arrows. GC, guard cell; GMC, guard mother cell; M, meristemoid cell; MMC, meristemoid mother cell; PC, protodermal cell; YGC, young guard cell. Scale bar =50 μm in UV and Bright field.

To gain an in-depth understanding of stomata development, we further identified several sets of genes critically involved in the regulation of the cellular processes. Using protein sequences of the essential stomatal development regulators to search the tea plant genome, we were able to find almost all major regulatory components with functions in regulating stomatal lineage [23]. AtEPF1/2 homologs in the tea plant genome included *CsEPF1/2* (TEA008903) (Fig. 3A). The tea plant homologs for Arabidopsis LRR-receptor like receptor kinase complex AtER-AtERL1/2-AtTMM included *CsER* (TEA016781), *CsERL1/2* (TEA002922), and *CsTMM* (TEA013511) (Fig. 3A). They are highly expressed in tea leaves (first, second, and third leaf), suggesting that *CsER*, *CsERL1/2*, and *CsTMM* might be involved in the perception of EPF signals for regulating stomatal development in tea plants. The AtEPFL9 (STOMAGEN) homolog gene in tea plants *CsEPFL9* (TEA010174) was highly expressed in all developing leaves, indicating a probable important role in stomatal development in tea plants (Fig. 3A).

As a negative regulator in stomata development, Arabidopsis AtSDD1 mutants led to multiplication of stomata on both abaxial and adaxial epidermis in leaf surface. AtSDD1 homolog gene in tea plants, *CsSDD1* (TEA015301), was also expressed in an increasing trend, opposite to these positive stomata regulator genes (Fig. 3A). *CsSDD1* expression reached the highest level in the second leaf, perhaps inhibiting the stomatal development (Fig. S2 and Tables S2–3, see online supplementary material).

During stomatal development, EPFs bind to ER-ERL-TMM receptor kinases, which activate their own kinase activity, to phosphorylate downstream MPKKKs. Their tea plant homolog genes included *CsYODAa* (TEA027265) and *CsYODAb* (TEA008165), MAPKK genes *CsMAPKK4* (TEA001048), *CsMAPKK5*(TEA007966), *CsMAPKK6a* (TEA015514), *CsMAPKK6b* (TEA025635), *CsMAPKK7a* (TEA008204), and *CsMAPKK7b* (TEA007510), as well as *CsMAPK3*(TEA026040), *CsMAPK6*(TEA024415) (Fig. 3A). Most of these protein kinase genes were expressed at higher levels in apical buds and young leaves than in old leaves, similar to their counterpart genes in Arabidopsis plants (Fig. S2 and Tables S2–3, see online supplementary material).

As the direct downstream target of MPKs, four critical Arabidopsis bHLH regulators also had counterparts in tea plants, such as *CsSPCHa* (TEA029105), *CsSPCHb* (TEA029434), *CsMUTE* (TEA025673), *CsFAMA* (TEA011370), *CsSCRM/CsICE* (TEA009785), *CsSCRM2a*(TEA000036), and *CsSCRM2b* (TEA013512) (Fig. 3A). They displayed similar gene expression patterns in tea plant developing leaves with these in Arabidopsis leaves. Tea plant MYB regulators involved in stomata development, such as *CsMYB88* (TEA019308), *CsMYB124b* (TEA025556) and *CsMYB124a* (TEA007744) also had similar gene expression patterns in tea plant developing leaves with these in Arabidopsis leaves. There, genes were mainly expressed in apical buds and young leaves, in which epidermis cells in active developmental stages into stomata (Fig. S2 and Tables S1–2, see online supplementary material).

Furthermore, homolog genes involved in cell division and guard cell formation were also obtained from the tea plant genome by BLAST. The homolog genes for AtCYCDs and AtCDKA/B, including *CsCYCD7-1a* (TEA008391), *CsCYCD7-1b* (TEA022072), *CsCYCD3-1a* (TEA025845), *CsCYCD3-1b* (TEA014197), *CsCYCD3-2a* (TEA004537), *CsCYCD3-2b* (TEA008197), *CsCYCD5-1a* (TEA029991), *CsCYCD5-1b* (TEA011528), *CsCYCD5-1c* (TEA031243), *CsCDKA-1* (TEA011791), *CsCDKB1–1* (TEA019056) and *CsCDKB1–2*(TEA01 8325) displayed the highest expression levels in apical buds, decreasing over the leaf development. The homolog genes regulating cell cycle for AtMYB3R, AtSOL1, and AtSOL2, including *CsMYB3R-1*(TEA024836), *CsMYB3R-4* (TEA024305), *CsSOL1* (TEA025162), *CsSOL2a* (TEA022204) and *CsSOL2b* (TEA031233) were also expressed in young leaves and then their expression levels decreased (Fig. S2 and Tables S1-2, see online supplementary material). The expression patterns of these genes were consistent with their function in early PC stomatal lineage formation.

### Variations in stomatal density and developmental rate in different tea plant varieties

**Figure 4:**
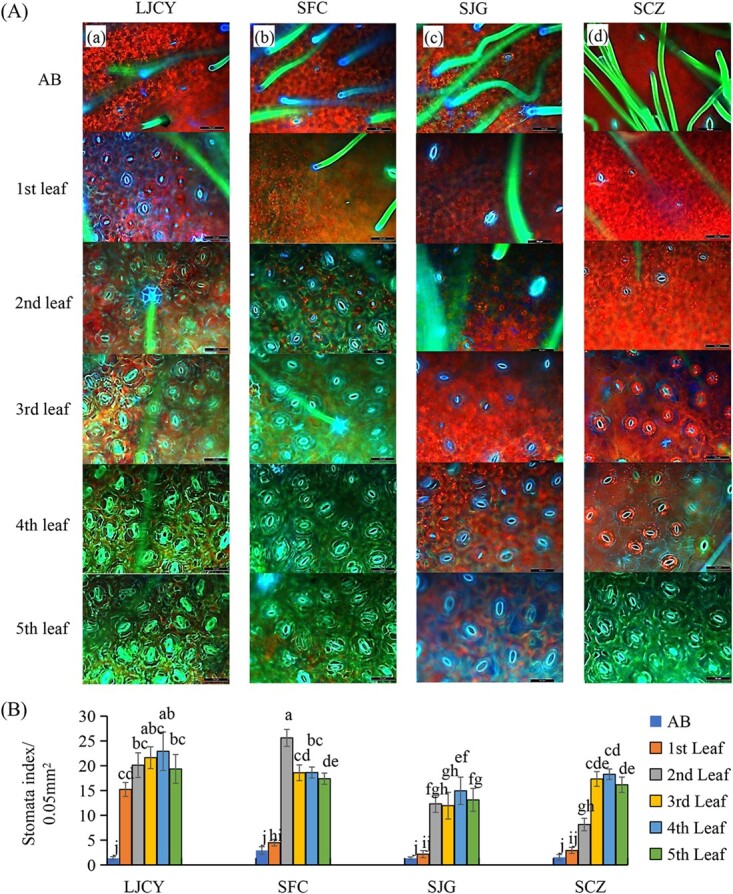
Variations of stomatal density in tea plant varieties. **A** The images of stomata and trichomes in the apical bud (AB), the first, second, third, fourth and fifth leaf of LJCY (a), SFC(b), SJG(c), and SCZ(d). Scale bar = 50 μm (×40). LJCY and SFC have relatively higher stomatal densities on their leaves, whereas SJG and SCZ had the lowest ones. **B** Comparison of stomatal densities in developing leaves [the apical buds (ABs), the first, second, third, fourth and fifth leaf of various tea plant varieties]. Data were obtained from three independent experiments with triplicate, and multiple comparisons with the ANOVA multiple range test at the 0.05 probability level. The stomatal density was expressed as stomata/0.05 mm^2^. The tea plant varieties are LJCY, Longjingchangye; SCZ, Shuchazao; SJG, Shuijingui; SFC, Shifocui.

To understand how stomatal density and size vary in different tea plant varieties, we tested the stomata of various developing leaves from 14 tea plant varieties, which are of the same age and grown in a tea garden in similar environments and conditions (Jinzhai, Anhui). The stomata densities and sizes of different tea varieties varied significantly. However, the stomata number changes followed the same trends in all varieties: stomata number increased over the leaf development and mostly reached the maximum at third or fourth leaf, then decreased as leaf areas expanded but no new stomata formed in mature leaves (Fig. 4A).

To further clarify the molecular basis underlying the stomatal developmental trend and validate their expression patterns in developing leaves, we chose four tea varieties that showed extreme stomatal density and size among these 14 tea varieties. Two varieties, ‘Longjingchangye’ (LJCY) and ‘Shifocui’ (SFC) had the highest stomatal densities but smaller stomatal sizes, whereas ‘Shuchazao’ (SCZ) and ‘Shuijingui’ (SJG) had the lowest stomatal densities and biggest stomata among these 14 tea varieties (Fig. 4A). The distinguishable variations in stomata density and size in developing leaves of different tea plant varieties were observed and recorded under a fluorescence microscope.

The apical buds of these tea varieties were covered with trichomes, showing green pseudocolor by UV light excitation under the fluorescence microscope. While trichome number decreased over leaf development, stomata number increased rapidly until reaching the peak in the third to fourth leaf. With the highest stomatal density, LJCY had the stomata number suddenly increased in first leaf as compared with these in AB, and even in first leaf there are many potential small stomata. However, in SFC developing leaf, the significant stomata number increase occurred at the second leaf as the stomata number was still low in the first leaf and AB. The stomata density in SJG and SCZ were lower, and obviously increased at the third leaf as compared with the low number and slow development of stomata in AB, first and second leaf (Fig. 4B; Fig. S3, see online supplementary material). The density of stomata in SJG and SCZ also gradually increased and reached the maximum in the fifth leaf. However, their stomata spacing became larger than those in LJCY and SFC (Fig. 4A) (Fig. S6, see online supplementary material).

### Expression of key bHLH and MYB regulator genes in tea stomatal development

**Figure 5:**
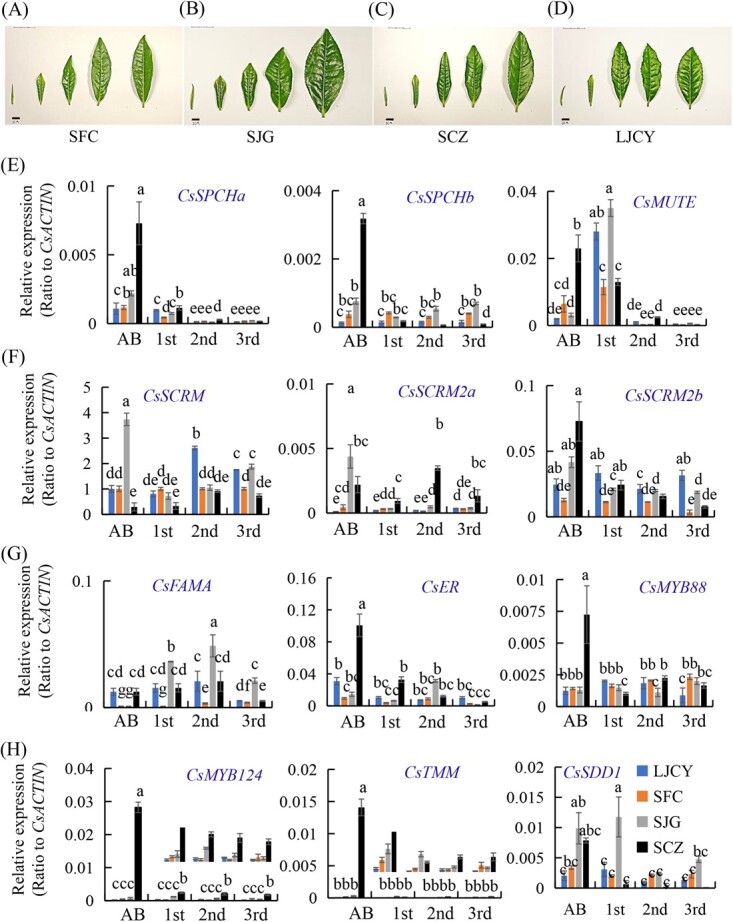
Involvement of *bHLH* and other genes in stomatal development in four tea plant varieties. **A**–**D** Shoot tips of tea plant varieties Shifocui (SFC) (**A**), Shuijingui (SJG) (**B**), Shuchazao (SCZ) (**C**), Longjinchangye (LJCY) (**D**). **E** Expression patterns of *CsSPCHa*, *CsSPCHb*, and *CsMUTE* in the apical bud (AB) and first, second, and third leaf of four tea plant varieties. **F** Expression patterns of *CsSCRM, CsSCRM2a*, and *CsSCRM2b* in the apical bud (AB) and first, second, and third leaf of four tea plant varieties. **G** Expression patterns of *CsFAMA, CsER*, and *CsMYB88* in the apical bud (AB) and first, second, and third leaf of four tea plant varieties. **H** Expression patterns of *CsMYB124*, *CsTMM*, and *CsSDD1* in the apical bud (AB) and first, second, and third leaf of four tea plant varieties. The relative expression levels of the mRNA genes were measured via qRT-PCR and normalized to the average expression level of *CsACTIN*. Data are expressed as the means ± SD from three independent experiments, each with multiple biological replicates. The differences between tea plant varieties were analysed with two-factor ANOVA using the LSD0.05 method.

To further understand functions of these genes in stomatal development and validate their expression patterns in developing leaves, we examined these four tea varieties that showed different stomata lineage outcomes, such as development rate, stomatal density and size. *CsSPCH*, *CsMUTE*, *CsFAMA*, *CsER*, *CsMYB88*, *CsICE1*, *CsSCRM2*, *CsTMM* and *CsMYB124* genes were highly expressed in shoots and young leaves, among eight different tissues (Fig. S2; Tables S1-2, see online supplementary material). As SPCH is the core regulator of stomatal density, SPCH is expressed in epidermal cells, and its protein is accumulated in MMCs and MCs [24]. *CsSPCHa* showed the highest expression level in apical buds and then gradually decreased over leaf maturation, so did *CsSPCHb*, while *CsSPCHa* and *b* were expressed at higher levels in SCZ and SJG, about 3-fold higher in SCZ than in others (Fig. 5E). *CsSPCHa* and b expression levels seemed to be opposite to the stomatal density variation.

MUTE regulates the transition of MCs into GMCs, as evidenced by arrested stomatal lineage at the MC stage in Arabidopsis *mute* mutants [20, 24]. The canonical *CsMUTE* in tea plants was expressed at similar levels in different cultivars, with a peak in the first leaf and then decreasing rapidly over the maturation of leaves (Fig. 5E). This indicated that the end of the asymmetric division in stomatal lineage and the appearance of GMC in the first tea leaf. FAMA regulates the last symmetric cell division of each GMC into a pair of GCs to form stomata and thus terminates lineage cell meristematic activity [15, 20, 24]. *CsFAMA* is also a single-copy gene in the tea plant genome. *CsFAMA* transcript levels were at their highest levels at first and second leaves, where most GMCs developed into GCs, then decreased to the lowest levels in mature leaves [25, 26]. The first and second leaf of SJG had the highest expression level but the lowest stomatal density and larger stomatal size. SFC showed the lowest *CsFAMA* expression level, consistent with the highest stomatal density but smaller stomatal size. In consistency to its function, *CsFAMA* transcripts reached the highest level at the second and third leaf and then decreased to the lowest in the mature leaves (Fig. 5G). *CsSCRM/ICE1* restricts symmetric divisions, ensuring that stomata contain a pair of GCs; *CsSCRM/ICE1* and *CsFAMA* share a common expression window in stomatal development [8, 15, 24]. *CsSCRM* transcript level increased continuously over the leaf development, while the expression level of SJG was the highest in AB and then gradually increased (Fig. 5F).

*CsSCRM2a* and *CsSCRM2b* were expressed at higher levels in apical buds than in other leaves. Apparently, they were expressed at higher levels in lower stomata-density varieties, such as SCZ and SJG, but at lower levels in high-stomata density varieties LJCY and SFC. The overall trend of *CsMYB88* transcripts increased first and then decreased with the development of leaves [25]. The expression of *CsMYB124* in different leaf development in SCZ was the largest with the growth of leaves, and the change was the largest during the process from AB to leaf, and the expression of *CsMYB124* decreased slowly in the other three varieties [26]. The level is high when first leaf and second, then decreases (Fig. 5H). As the early stomata lineage gene, *CsER* was expressed at the highest level in apical buds, and then the expression level rapidly decreased over leaf development. The low-stomata density tea varieties SJG and SCZ have the highest *CsER* expression levels, much higher than those in high stomata density varieties LJCY and SFC (Fig. 5G). *CsER* is related to the natural growth state, the low expression of *CsER* help maintain normal stomatal lineage base cells (SLGC).

Despite the importance of TMM in Arabidopsis, the expression level of *CsTMM* in tea leaves was very low, with a highly similar decreasing trend from apical bud to mature leaf. SJG and SCZ have the highest *CsER* expression levels (Fig. 5G), particularly in SCZ with the highest expression level. The high stomata density varieties LJCY and SFC had much lower *CsTMM* expression levels than SJG and SCZ (Fig. 5H). While SPCH is the core regulator of stomatal density, TMM delays the MC to GMC conversion by prolonging MC fate [7, 19, 24]. TMM is a positive regulator of MCs in both leaves and stems and suggests that the role of TMM in promoting MC division in leaves is separable from its essential role in promoting the developmental progression of MCs in stems [27, 28]. It was found that the expression level of *CsTMM* was high in tea plant buds and very low in leaves, but the change trends for the four varieties were highly similar. As a negative regulator, Arabidopsis *SDD1* is expressed predominantly in specialized epidermis cell types, and its mRNA was not detected in mature GCs [9, 16]. *SDD1* overexpression caused significantly decreased stomatal density and formation of arrested stomata at the M/GMC stage, whereas the mutation of *SDD1* gene led to the multiplication of stomata on both abaxial and adaxial epidermes [9, 16, 24].

In comparisons of four tea plant varieties with significantly different stomatal densities, *CsSDD1* was generally expressed at lower levels in LJCY and SFC, whose leaves contained higher stomatal densities (Fig. 5H). By contrast, *CsSDD1* was expressed atmuch higher levels in SCZ and SJG, more than 2-fold higher than in LJCY and SFC; and SCZ and SJG’s leaves contained lower stomatal densities (Fig. 5H). These data strongly suggested that CsSDD1 in tea plants also negatively regulated leaf stomatal density and the suppression of CsSDD1 on leaf stomata density, likely through the formation of arrested stomata at the M/GMC stage, as we have observed.

### Effects of light conditions on stomatal development in different tea plant varieties

**Figure 6:**
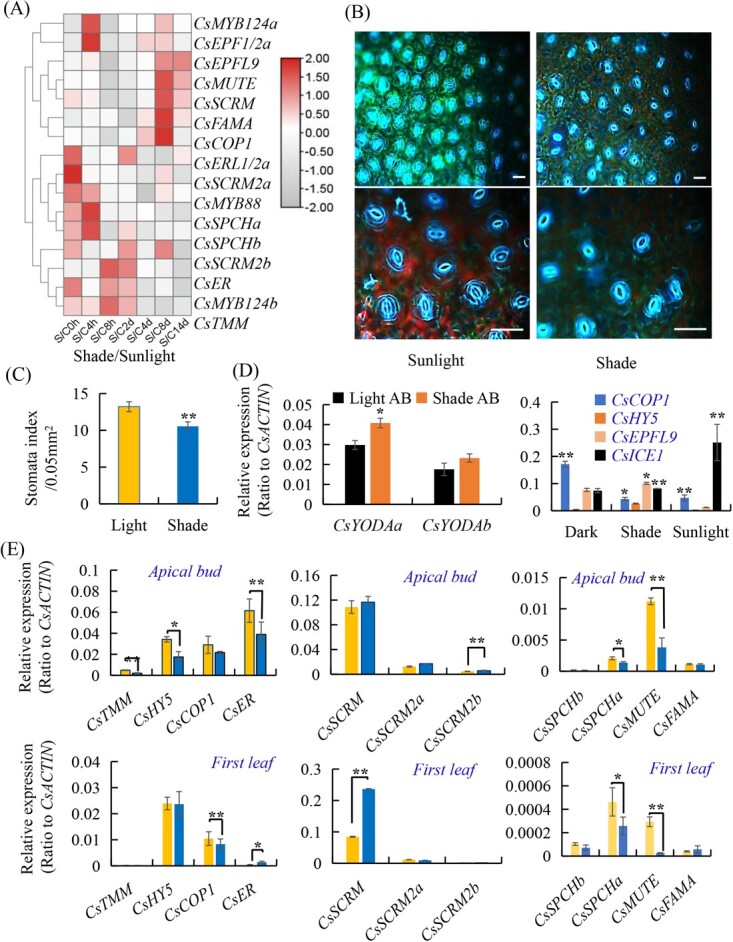
Regulation of stomatal development in tea leaves by light conditions. **A** Heatmap analyses of stomatal lineage gene expression under regular sunlight and shading treatment. **B** Stomata density in abaxial tea leaves under regular sunlight or shading treatment. Light exposure increased the stomata number per unit leaf area, whereas shading treatment reduced stomata density of tea leaves after 14 days of shading treatment. The stomata are imaged under a fluorescence microscope. Scale bar = 50 μm. **C** Quantification of stomata in tea leaves under different lighting conditions. Stomata density on leaves was reduced after the long-time of shading treatment as compared with regular sunlight conditions. **D** Expression of *CsYODAa*, *CsYODAb* in the apical bud (AB) and *CsHY5, CsCOP1, CsEPFL9*, and *CsSCRM/ICE1* in young leaves under sunlight, shading, and the dark for 12 hours by qRT-PCR. **E** Expression of stomata lineage genes in the apical bud and first leaf under sunlight or shading treatment. Tea plants under shading treatment with only 10–20% sunlight intensity (50 μmol m^−2^ s^−1^), compared with regular sunlight (350 μmol m^−2^ s^−1^). Comparative gene expression of tea light and shade treatments shoot tips (AB) was analysed with qRT-PCR. Data were expressed as the means ± SD from three independent experiments with triplicate and analysed using the Student’s *t*-test in a two-tailed comparison (^*^P < 0.05 and ^**^P < 0.01).

Light significantly influences the development of stomata, and light can promote stomatal clustering, and increase the stomatal size and density [29, 30, 31, 32]. To test whether light is involved in stomatal development in the tea plant, we analysed transcriptome data of the shading treated tea leaves, with regular sunlight as control [30]. Results showed that the gene expression levels of stomatal development-related genes *CsSPCHa*, *CsSPCHb* and *CsSCRM2a*, *CsSCRM2b*, *CsERL1/2a*, *CsMYB88*, *CsER*, *CsMYB124b* and *CsTMM* were all decreased under 14 days of shading treatment (Fig. 6A). While *CsMUTE*, *CsFAMA*, and *CsSCRM/ICE1* were significantly down-regulated in 14 days of shading treatment, tea plant CONSTITUTIVE PHOTOMORPHOGENIC1 (COP1) homolog *CsCOP1* and *CsEPFL9* transcripts were increased after 8 days of shading treatment (Fig. 6A). Shading treatment also down-regulated *CsMAPKK4* and *CsMAPKK6a* after 14 days, but *CsMAPK6*, *CsYODAa*, *CsYODAb* transcripts increased in the eight days of shading treatment and then decreased in the 14 days of treatment. Transcript levels of *CsMAPKK7a*, *CsMAPKK7b*, *CsMAPKK6b*, *CsMAPKK3/5* increased in the first four days of shading treatment (Fig. S4; Table S3, see online supplementary material).

We observed the changes in stomata density on the leaves of tea plants under regular sunlight and shading treatments. The stomatal density of tea leaves under shade conditions was significantly lower than that under sunlight (Fig. 6B and C). To further understand the effect of light/dark on stomatal development, we quantitatively analysed the photo-regulatory response factors of *CsCOP1*. We found that *CsCOP1* was inhibited under light conditions, with the highest expression in the dark and decreased under light conditions. *CsSCRM/ICE1/* transcript was at the lowest level under dark conditions and increased with the increase of light intensity (Fig. 6D). *CsHY5* expression level was induced by sunlight conditions, and the expression level of *CsEPFL9* was higher under white light and in the dark, but lower under strong light (Fig. 6D) AtEPFL9 is responsive to light-activation of stomatal development, downstream HY5 [29]. Under light treatment, *CsEPFL9* was rapidly up-regulated with *CsbZIP1*, a *CsHY5* homolog in tea plants [29, 30], and under high-light treatment *CsHY5* was rapidly up-regulated.

Quantitative analysis of key genes regulating stomatal development in tea plants. The results showed that the expression of *CsSPCHa* in apical buds and first leaf under shading conditions was significantly lower than that in sunlight (Fig. 6E), which triggers more stomatal development and formation for carbon uptake in a light intensity-dependent manner through promoting SPCH accumulation [31, 32, 33]. The expression of *CsSPCHa* and *CsMUTE* in apical buds and first leaf was significantly lower under shading than those in sunlight. The *CsSCRM* and *CsER* transcript levels were significantly higher in the first leaf in shading than under sunlight. *CsSPCHb* and *CsFAMA* had no significant difference between the two conditions. The expression level of *CsTMM2*, *CsHY5*, *CsEPF1/2* in apical buds in the sunlight were higher than under shading conditions (Fig. 6E). Also, *CsYODAa* was expressed at higher level in young leaves under shading than in sunlight (Fig. 6F; Fig. S4, see online supplementary material). The five genes, *CsSPCHa*, *CsMUTE*, *CsTMM*, *CsER*, and *CsEPF1/2,* were sensitive to light, and these data showed that these five genes could be inhibited under shading conditions to repress the development of stomata. These observations are also consistent with the reports that the expression of *STOMAGEN*, *SPCH, MUTE, FAMA, EPF2*, and *TMM* is induced by light in Arabidopsis [29, 31, 32, 33]. This is consistent with these factors as important regulators of stomata development, acting as regulatory hubs that environmental factors could target [7, 19, 24].

### Effects of temperature changes on stomatal development in different tea plant varieties

**Figure 7:**
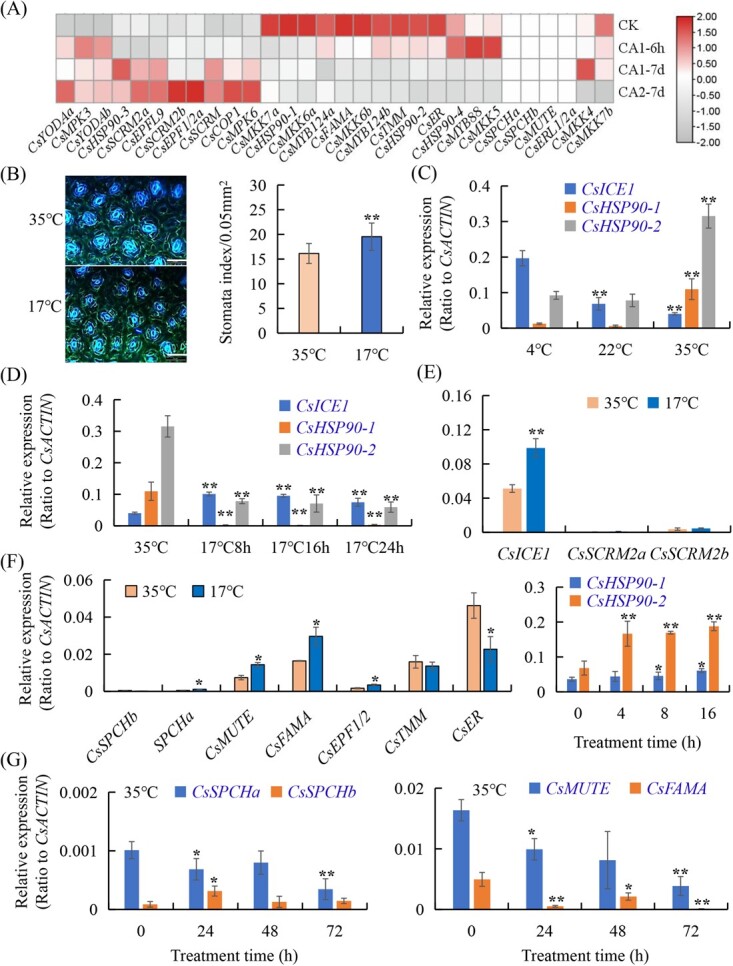
Temperature stresses regulated stomatal development in tea plant leaves. **A** Heatmap analysis of stomata lineage gene expression under cold treatments. CK: 25°C ~ 20°C; CA1-6 h: 10°C for 6 hours; CA1-7d: 10°C ~ 4°C for 6 days; CA2-7d: 4°C ~ 0°C for 7 days; DA-7d: recover under 25°C ~ 20°C for 7 days. **B** Stomata density changes in tea plant leaves under 17°C and 35°C. Left panel: Stomata images in tea leaves was observed under a UV fluorescence microscope. Scale bar = 50 μm. Right panel: quantification of leaf stomata density. **C** Expression patterns of the related genes in tea leaves under various temperatures. **D**–**F** The expression levels of stomata lineage genes and *CsHSP90*s under variable temperature conditions from 35°C to 17°C treatments. **G** Effects of heat stress (35°C) on the expression of *CsSPCH, CsMUTE,* and *CsFAMA* genes at different treatment time. Tea seedlings were incubated at different temperatures (4°C, 22°C, and 35°C) under consistent light conditions. The young leaves were used for gene expression assay via qRT-PCR and normalized to the average expression level of *CsACTIN*. Data are expressed as the means ± SD of three independent experiments, analysed by using the Student’s *t*-test in a two-tailed comparison (^*^P < 0.05 and ^**^P < 0.01).

Analysis of the transcriptome on cold-treated tea plant showed that *CsMAPK3*, *CsYODAb*, *CsMAPKK6*, *CsMYB88*, *CsSCRM2a*, *CsEPFL9*, and *CsHSP90–4* were up-regulated by cold treatment. *CsYODAa*, *CsSCRM2b*, *CsEPF1/2a*, *CsMAPK6*, and *CsCOP1* were up-regulated when temperature increased from cold treatment to room temperature. The expression levels of *CsMAPKK6a*,*7a*, *6b, CsMYB124a*, *b*, *CsHSP90–1*, *CsFAMA*, *CsER*, and *CsTMM* were repressed by cold treatment. While the expression levels of *CsSCRM*, *CsSCRM2a*, and *CsSCRM2b* increased when temperature recovered (Fig. 7A; Table S4, see online supplementary material), we found that low temperature treatment enhanced stomatal development and increased the stomatal number in tea plant leaves when temperature decreased to 17°C, as compared with these under 35°C (Fig. 7B). We also found that stomatal density in tea plant leaves grown under low temperature conditions (17°C) was significantly higher than that grown under high temperature, while sizes of stomata were much smaller than that of high temperature conditions (35°C) (Fig. 7B).

Stomatal differentiation and patterning are spatially and temporally regulated by a regulatory network composed of the master regulators SPCH, MUTE, FAMA, and SCRM [24, 30]. An Inducer of CBF Expression 1 (*ICE1*) is involved in regulating cold stress tolerance, and is allelic to *SCRM*, indicating that cold and high temperature affected stomatal development [34, 35, 36]. In order to further understand how tea stomatal lineage genes respond to temperature changes, gradient temperatures were set for tea plants in growth environments at 4°C, 22°C, and 35°C. *CsSCRM/ICE1* was significantly induced under 4°C, as compared with that at 22°C (Fig. 7C). In incubation at 35°C, the expression levels of *CsHSP90–1* and *CsHSP90–2* were significantly higher than that of control, indicating that *CsHSP90–1* and *CsHSP90–2* were up-regulated by 35°C heat stress (Fig. 7D and F). It was found that the expression of *CsICE1* of tea plants was the most significantly changed when transferring from 22°C to 4°C (Fig. 7C). Meanwhile, when the tea plants at high temperature (35°C) were transferred to a low temperature (17°C) environment, the expression of *CsSCRM/CsICE*, *CsSCRM2a*, *CsSCRM2b*, *CsSPCHa*, and *CsFAMA* was obviously up-regulated at 8 h after low temperature treatment (Fig. 7D), but *CsYODA* gene expression was up-regulated by high temperature (Fig. S5, see online supplementary material).

Under high temperature (35°C), the expression level of *CsTMM* became higher than that under low temperature. *CsER* expression was also significantly up-regulated by high temperature, but *CsSPCHa*, *CsMUTE*, *CsICE1/CsSCRM,* and *CsFAMA* were significantly suppressed by heat stress (35°C); *CsSPCHb* did not change significantly at low temperature (Fig. 7F and G), similar to that in Arabidopsis [36]. These results support that heat stress could lead to the repression of stomatal development in tea plant leaves, whereas low temperature could promote stomatal development.

Indeed, the expression patterns of stomata development- and movement-related genes often change in response to MeJA, drought, salt stress, and low temperature treatment. AtALMT9 and AtALMT6 are malate-activated vacuolar Cl^−^ channel and exclusively expressed in mesophyll cells and guard cells of Arabidopsis leaves, where they are involved in the export of Cl^−^ into the vacuole to regulate stomata movement [37]. The multidrug and toxic compound extrusion (MATE) transporters DTX33 and DTX35 are also highly expressed in Arabidopsis root hairs and guard cells that regulate turgor changes during root-hair elongation and stomatal movements [38]. There, homolog genes in tea plants also displayed similar expression patterns (Table S6, see online supplementary material). Upon MeJA treatment, *CsMYC2\4*, *CsALMT6*, *CsALMT9*, and *CsHSP90* were clearly up-regulated, whereas the expression of *CsSPCH*, *CsER*, *CsTMM*, *CsERL1/2* were down-regulated, *CsMUTE*, *CsFAMA,* and *CsSCRM* transcripts were induced initially and then recovered (Fig. S6A; Table S5, see online supplementary material). Under drought stress, *CsMYC2*, *CsALMT6*, *CsHSP90, CsSPCHb, CsMUTE, CsFAMA,* and *CsSCRM* expression levels decreased drastically. However, the transcripts of *CsALMT9*and *CsMATE* increased significantly after 24 h of treatment (Fig S6B; Table S6, see online supplementary material). Under cold treatment, *CsHSP90–3*, *CsMYC4* and *CsSCRM* were up-regulated (Fig. S6C; Table S4, see online supplementary material). Under NaCl treatment, *CsMYC4*, *CsALMT9*, *CsMATE*, *CsHSP90–3,* and *CsSCRM2b* were up-regulated, whereas *CsHSP90–1*, *CsHSP90–2*, and *CsMYC2* were repressed, so did stomata lineage genes *CsSPCH*, *CsMUTE*, *CsFAMA*, and *CsSCRM* (Fig. S6D; Table S7, see online supplementary material). Among four tea plant varieties, SJG and SCZ leaves had higher water loss rates and larger stomatal apertures than LJCY and SFC leaves. This is also consistent with lower stomatal densities in SJG and SCZ than in LJCY and SFC, indicating that negative correlation between the stomatal density and stomata-mediated water loss rate. Stomata apertures were positively correlated to water loss rate and determined the leaf water loss in tea plants (Fig. S7, see online supplementary material).

We further examined the dynamic stomata movement and function of SCZ leaf during the daytime. Most stomata opened in the morning, reached maximal stomata aperture at 11:00 and closed when it reached the highest temperature and strongest sunlight intensity at noon (Fig. S8A, see online supplementary material). Then, stomata conductance and photosynthesis rate showed a downward trend with the so-called ‘photosynthetic midday depression’ (Fig. S8B–E, see online supplementary material). LJCY, SFC, SJG, and SCZ simultaneous detection of stomatal conductance, net photosynthetic rate and intercellular CO_2_ concentration in LJCY, SFC, SJG, and SCZ leaves found that they showed similar trends except for the different intercellular CO_2_ concentration with SCF cells (Fig. S8F–H, see online supplementary material). Photosynthesis analysis showed that net photosynthesis rate, stomatal conductance, and intracellular CO_2_ concentration in SFC and LJCY were higher than those in SCZ and SJG. Thus, these parameters seemed positively correlated with stomatal density (Fig. S8F–H, see online supplementary material). Thus, stomata movement and density directly affect the net photosynthetic rate and intercellular CO_2_ concentration of tea plants**.**

### Gene expression underlying different stomata patterning in tri- and di-ploid tea plants

**Figure 8:**
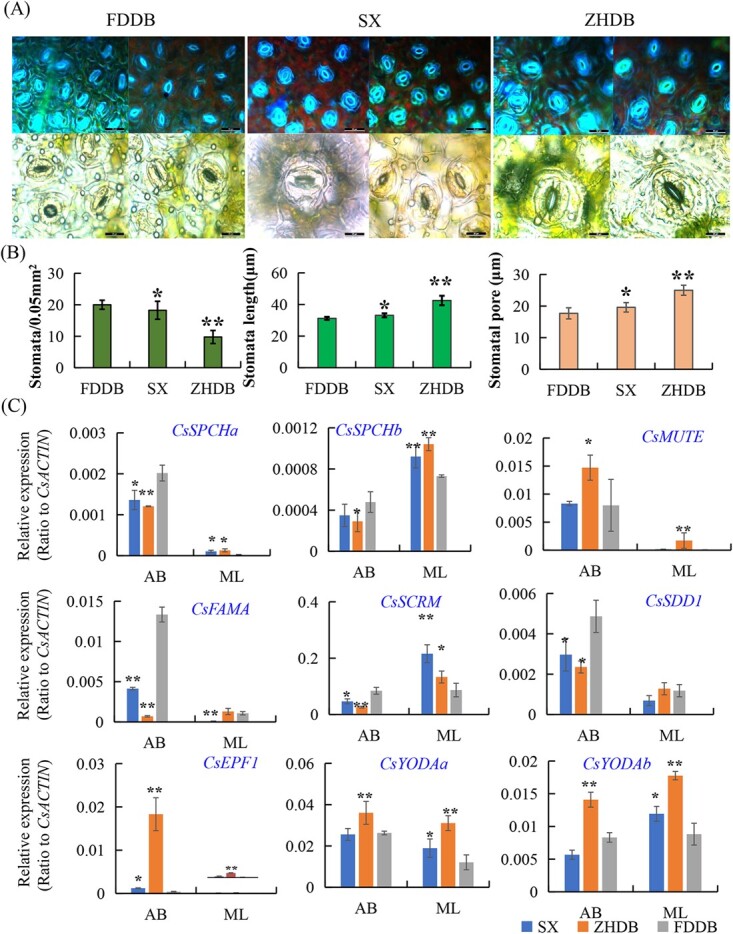
Altered stomatal phenotypes in triploid tea plants and underlying gene expression variations. **A** Morphological differences in stomata of triploid tea varieties ‘ZhenHeDaBai’ (ZHDB) and ‘ShuiXian’ (SX) and a diploid tea variety ‘FuDingDaBai’ (FDDB). Stomata in mature leaves were imaged under a fluorescence microscope with UV-light (top panel, bar = 50 μm) and bright-field (bottom panel, bar = 20 μm). (**B**) Quantitative comparison of stomata density and size between triploid tea varieties ZHDB and SX and a diploid tea variety FDDB. Image J software was used for the measurement of stomatal size and density. **C** Comparison of gene expression profiles in apical buds (AB) and mature leaves (ML) between triploid tea varieties ZHDB and SX and a diploid tea variety FDDB. Relative gene expression was tested with qRT-PCR. Data are expressed as the means ± SD from least three independent experiments, and statistical analysis by using the Student’s *t*-test in a two-tailed comparison for the significance, ^*^P < 0.05 and ^**^P < 0.01.

Plants are usually able to optimize CO_2_ uptake for photosynthesis while minimizing water loss by regulating stomatal density, size, and movement [4, 5, 6]. Genetic manipulation of stomatal density to improve plant productivity and water use efficiency has been proven to be feasible in barley and rice [9, 10]. It is known that plant polyploidy could alter photosynthesis capability and stomatal development while increasing plant genome size [39, 40, 41]. We further examined the stomata phenotypsphenotypes, stomata density, and size of two well-known triploid tea plant varieties (3x = 45), ‘ZhenHeDaBai’ (ZHDB) and ‘ShuiXian’ (SX), in comparison with regular diploid tea varieties [42]. Under identical conditions, the mature leaves of ZHDB had significantly lower stomata densities but larger stomata sizes than a representative diploid tea ‘Fudingdaba’ (FDDB), but SX leaves displayed clearly mixed stomatal densities and sizes: some are similar to these of ZHDB with lower density but larger stomata, but others are similar to these of FDDB with smaller stomata but in higher density (Fig. 8A; Fig. S9, see online supplementary material). Images of the stomata in these plant leaves can easily describe the quantitative differences in stomatal density and size (Fig. 8B; Fig. S9, see online supplementary material). To understand genes altered in expression that could be related to the stomatal phenotypes, we further conducted qRT-PCR to examine several key genes involved in tea plant stomatal development (Fig. 8C). As positive regulator genes, both *CsSPCHa* and *CsSPCHb* were expressed at higher levels in apical buds of FDDB than in ZHDB and SX, but displayed opposite patterns in mature leaves. *CsSCRM/ICE1* and *CsFAMA* were expressed in similar patterns to *CsSPCH*s, which are consistent with higher stomatal density in diploid but larger in triploid varieties. Only *CsMUTE* showed slightly higher expression levels in ZHDB than in FDDB and SX (Fig. 8C). Three negative regulator genes, *CsYODAa* and *CsYODAb*, and *CsEPF1*, were expressed at higher levels in triploid tea variety ZHDB than in diploid FDDB and another triploid SX, which were also consistent with their stomatal density and size phenotypes (Fig. 8C). Although *CsTMM* and *CsER* were expressed higher in diploid FDDB than in triploid ZHDB and SX (Fig. 8), TMM and ER can serve as molecular markers for stomata precursor cell development in feedback loops behind their functions [7, 19, 24].

## Discussion

### Tea plants have consecrated stomatal development and regulatory mechanisms

Stomatal density, spacing, and patterning influence the efficiency of gas exchange, photosynthesis, water usage, stress response, and disease resistance, and other aspects of plant life [1, 2, 3]. In spite of this importance, the information about stomata development and movement in tea plants are not reported so far, as tea plants are adapted from tropical and subtropical regions, but their territories are expanding further north. Therefore, it is quite interesting to know tea leaf stomata and their functions. Similar to Arabidopsis, tea leaf stomatal development could also be regulated by similar signaling components, including EPF1/2, TMM, and ER and ERL1/2, a MAP kinase cascade, and downstream TFs of various types to determine cell fate and stomata formation. The patterning of leaf stomata is regulated by EPF1, EPF2 and STOMAGEN, and bHLH TFs, MAP kinase cascade, and cell cycle proteins [43, 44, 45, 46]. The similarity of tea plant CsEPF1/2 and STOMAGEN with Arabidopsis counterparts at protein sequence and gene expression levels is high. In our observation of tea leaf stomata developmental patterns, the stomata number increases coincided with some of these regulator gene expressions in developing leaves.

Despite the similarity, SPCH, MUTE, and FAMA are functionally distinctive from each other during stomatal development [7, 41]. SCRM and SCRM2 redundantly bridged SPCH, MUTE, and FAMA activities through heterodimerization [15, 41]. FLP and MYB88 also redundantly restrict GMC cell division and promote the stomatal transition from GMC to GCs [16,42–44]. FAMA and FLP/MYB88 act in parallel to restrict GMC division, through CDKB1;1, together with CDKB1;2 [20, 25, 26, 42, 45, 46]. Their tea plant homolog genes followed similar expression patterns in tea leaf developmental stages (Fig. 2N and O; Fig. S2, see online supplementary material). Moreover, the positive or negative regulatory functions of their homolog genes in tea plants were also shown in trioloid tea varieties with lower stomatal density and size. *CsSPCH, CsSCRM, CsFAMA* were all expressed at higher levels in diploid tea plant than triploid tea plants, in consistence with their roles in promoting stomatal formation. The expression patterns of these key genes are consistent with stomatal development and stage transitions. For instance, *CsSPCH* was highly expressed in apical buds, *CsMUTE* showed the highest expression at the first leaf, whereas *CsFAMA* was highly expressed in young leaves (from the first to third leaf) (Fig. 5).

### Light regulator stomatal development in tea plant

Light is one of the most important factors that critically regulate plant growth and development. Light regulation stomata development has been well-documented [29, 31, 32, 33]. Only a few mature stomata formed occasional stomatal clusters in the dark-grown Arabidopsis, but light enhances the division of MMC into M and then the division of GMCs to form mature stomata [31, 32]. Therefore, light perception and signal transduction essentially shape the plant stomata development. The formation of MC and stomatal maturation is defective in photoreceptor mutants (*phyB, phyA*, and *cry1cry2*) [34]. In our study, we also observed that expression of *CsSTOMAGEN*, *CsSPCH, CsMUTE*, *CsFAMA*, and *CsTMM* homolog genes was induced by light in tea plant buds (Fig. 6) [30]. COP1 is a central negative regulator of photomorphogenesis acting downstream of the PHY and CRY photoreceptors [33, 34]. COP1 and TMM/ER/ERL-mediated signaling converged to YODA to suppress stomata formation by promoting phosphorylation and subsequent degradation of SPCH and SCRM [31, 32, 34]. In tea plants, regular light promoted stomata development, whereas shading treatment reduced stomata numbers. Regularlight activated tea plant stomata development regulator genes, such as *CsSTOMAGEN/CsEPEL9, CsSPCH, CsMUTE, CsFAMA,* and *CsSCRM/CsICE1*. Meanwhile, regular light inhibited the negative regulator genes, such as *CsER, CsCOP1*, and *CsYODA* (Fig. 6).

### Effects of temperature stomatal development in tea plant

HEAT SHOCK PROTEINS 90 (HSP90) is a molecular chaperone playing an important role in transducing heat-stress response to leaf stomatal development via modulating the YODA kinase cascade [24, 47]. HSP90 interacted with and affected YODA cellular polarization, and modulated the phosphorylation of downstream targets, such as MAPK6 and SPCH, under heat-stress conditions [24, 35, 47]. HSP90.1 and HSP90.2 had different roles in the adaptation of stomatal development to heat stress, and HSP90s and MAPKs play a prominent role in the destabilization and transcriptional deactivation of *SPCH*, and lead to the repression of stomatal development [24, 35, 36, 47]. Our study showed that *CsHSP90*s, *CsYODA, CsTMM,* and *CsER* were induced, whereas *CsSPCHa*, *CsMUTE*, *CsICE1/CsSCRM,* and *CsFAMA* were significantly suppressed by heat stress (Fig. 7) [47].

SCRM/ICE1 binding to the promoters of *CBF* genes and other regulatory genes is known to be critical for the cold response as well as to the activation of the promoters of some *COR* genes [24, 35, 36, 48, 49]. However, it is not understood the mechanism coordinating these actions. We identified three homolog proteins in tea plants, *CsSCRM*, CsSCRM2a, and 2b, which could resemble Arabidopsis counterparts to interact with and specify the sequential actions of CsSPCH, CsMUTE, and CsFAMA [3, 15, 34]. The gain-of-function mutation in *ICE1/SCRM* exhibited constitutive stomatal differentiation in the epidermis [6, 8, 15]. Conversely, successive loss of *SCRM/ICE1* and *SCRM2* recapitulated the phenotypes of *fama*, *mute,* and *spch*, indicating that they determined successive initiation, proliferation, and terminal differentiation of stomatal cell lineage [6, 8, 15]. SPCH, MUTE, and FAMA heterodimerize with SCRMs to trigger the successive MMC-M-GMC-GC fate transition [15]**.** A recent study demonstrated that PIF4 played a critical role in the stomatal development in response to high temperatures [36]. The heat-activated PIF4 can bind and repress *SPCH* expression to restrict stomatal production [36].

### Triploid tea plants developed larger but fewer leaf stomata due to altered gene expression

Water loss through stomata usually depends on many factors, such as density, size, stomata functions, and regulatory integrity, upon water deficit or dehydration stress [9, 13, 48]. Manipulation of genetic factors affecting water loss rate through stomata has provided insight into the genetic improvement of plant water-use efficiency and drought tolerance [9, 10, 49, 50, 51]. It has been reported that triploidy or tetraploidy decreases stomatal density but increases stomata size in some plant species [39, 40, 41]. Similar to other plants [40, 41], we also observed that triploid tea plant varieties have larger stomata sizes but lower stomata density in developing or mature leaves at corresponding developmental stages. Although a correlation between stomata density and size and genome size has been documented [9, 13, 39, 40, 41], the genetic and molecular mechanisms regulating stomata density remain elusive. The overexpression of *AtEPF2, OsEPF2,* or *PdEPF1* in Arabidopsis or poplar plants resulted in particularly low stomata density in transgenic plants [9, 50, 51]. The overexpression or elimination of *SDD1* also leads to a significantly decreased or increased stomata density in Arabidopsis [51]. Interestingly, we found that although most key bHLH TF genes were expressed at higher levels in diploid tea than in triploid tea varieties, and these key negative regulator genes such as *CsYODA*s and *CsEPF1* were expressed at significantly higher levels in triploid tea ZHDB than in FDDB, as expected, the expression patterns of negative regulator genes, *CsSDD1, CsTMM,* and *CsER*, however, were not consistent with what expected. In perennial polyploid trees, enlarged vegetative tissue or organ phenotypes could partially be explained by dose-dependent gene expression. However, tea plant genomes have generally higher heterozygosity [22, 23]. Previous studies showed that triploid tea plants contain heterogeneous cells of genome size (e.g. about 30.0% ~ 56.9% ZHDB leaf cell are triploidy [42]). The even higher heterozygosity or heterogeneous cells in SX leaves may explain why SX showed such a huge diversity in stomatal morphology, mixed densities and sizes, and observation. The polyploid plants usually have larger leaf epidermis cells, higher stomatal conductance, higher photosynthesis rate, and higher water use efficiency [9, 13, 39, 40, 41].

In summary, this study demonstrated for the first time that stomatal development in both morphological change and molecular regulation context, under either regular developmental cures or different environmental conditions, such as light/shade, high temperature or cold stress, and polyploidy for stomatal density and size variations in comparison with diploid tea plants. Analyses of stomatal lineage genes under stresses and polyploid genetic backgrounds, the key tea plant genes regulating stomatal development or determining stomatal density and size were unveiled. Thus, by conducting morphological observations of epidermis protodermal cells to guard cell formation, identifying and profiling the expression patterns of stomatal lineage genes, and characterizing the critical stomatal lineage genes in regulation of stomata formation, density, and size in the triploid background and response to light and temperature changes, our study provides first new insight into tea plant stomatal development and regulation. This study lays a foundation for further exploring the genetic improvement of water use efficiency in tea plants to live up to challenging global warming and climate changes.

## Materials and methods

### Plant materials, growth conditions, and various treatments

*C. sinensis* (L.) O. Kuntze cv. ‘Shuchazao’, and other tea varieties are grown at the tea plantation of Anhui Agricultural University, Hefei, Anhui Province, China, Tea Garden at JinZhai (JinZhai County, Anhui province). The apical buds and leaf samples were taken in four seasons within 2 years (2020–2022) for phenotype survey and transcriptome analyses. The triploid tea plant varieties *C. sinensis* ‘ZhenHeDaBai’ (ZHDB) and ‘ShuiXian’ (SX), and a diploid tea variety ‘FuDingDaBai’ (FDDB) with similar background were grown in Tea Institute of Hunan Academy of Agricultural Science, Changsha, China, and apical buds and leaves of various developmental stages were sampled for stomatal observation and RNA analyses. For temperature stress on tea seedlings, the 2.5-year-old tea seedlings were treated in a growth chamber set at 4°C, 35°C, and 22°C (as a control) with regular 8/16 h dark–light period for various time. The shading experiments, methyl jasmonate (MeJA) treatments, polyethylene glycol (PEG) and NaCl treatments, the cold treatment experiments, were described previously [52, 53, 54, 55]. The normalized transcriptome data were retrieved from the Tea Plant Information Archive (http://tpia.teaplant.org/index.html). For UV-B radiation and high-light treatment, the 1.5-year-old tea seedlings or 8-year-old tea plants in tea gardens for shading and sunlight treatments were conducted as described previously [30].

The stomata-dependent water loss experiments were conducted with detached leaves. The detached tea plant leaves from various tea varieties growing in tea gardens were put on the table with abaxial surface up to the open aeration and abaxial side down and sealed by the edge. At various times of treatment (0, 4, 8, and 16 h), leaf weight was measured and stomata were imaged. The stomata aperture was measured with Image J software (https://imagej.en.softonic.com/). These developing leaf materials were also collected and frozen in liquid nitrogen, and stored in a −80°C freezer for RNA analysis.

### Histochemical treatment

To facilitate more clear microscopy observation of leaf epidermis cells, the developing leaves were fixed in solution (acetate: ethanol = 1:7) for 8 hours [47]. The leaves were de-stained with 75% ethanol solution and then were observed under a fluorescence microscope under the white-light field. For observing and imaging tea leaf epidermis cells, a fluorescence microscope (LEICA DM3000S) was used under UV-light or white-light field. The stomata on the abaxial surface of the first leaf to fifth leaf and the first stem to the fifth stem from different tea varieties were observed. The stomata number in 0.05 mm^2^ leaf area was counted in more than 30 leaves of each tea plant variety by using Image J software. The observation and imaging of tea leaf epidermis cells were conducted with a water immersion objective and dry objectives. The images were analysed using Photoshop (LAS V4.12) software. CIRAS-3 Portable Photosynthesis System was used to read the value of tea tree third leaves photosynthesis and stomatal index.

### Photosynthesis assay

The photosynthetic gas exchange parameters, photosynthetic rate (Pn), intercellular CO_2_ concentration (Ci) and substomatal CO_2_ concentration (Ci) were measured using a CIRAS-3 portable photosynthesis system (PP Systems, USA). The light intensity, light quality (90% red, 5% blue, and 5% white light), CO_2_ partial pressure (Cr), relative humidity (100%), leaf temperature and LED (1200 μmol m^−2^ s^−1^) were set by an automatic control device in the CIRAS-3 photosynthesis system [56].

### Identification of tea stomata lineage genes

The sequences of stomata lineage genes and coding proteins were retrieved from the TAIR (https://www.arabidopsis.org/) and the Tea Plant Information Archive (http://tpia.teaplant.org/index.html), respectively [23]. The amino acid sequences were aligned using ClustalW, and MEGA 6.0 software was used to construct a phylogenetic tree by the NJ method with 1000 bootstrap replicates. The Pfam and SMART tools were used to identify conserved protein domains [52]. The sequence information is shown in Table S1 (see online supplementary material).

### Quantitative RT–PCR analysis of gene expression

Total RNA from developing tea leaves or treatment samples was extracted by using RNAprep Pure Plant Plus Kit (Tiangen, Beijing, China) according to the manufacturer’s instructions. RNAs quality was checked using a Thermo 2000 Bioanalyzer and an RNA NanoDrop ND-2000 Spectrophotometer (Thermo Fisher Scientific, Shanghai, China), and the first-strand cDNA synthesis was done with the Super SMART PCR cDNA Synthesis Kit (Clontech, Palo Alto, CA, USA). Quantitative real-time PCR (qRT-PCR) was conducted using the SYBR Green PCR products (Yushen, Shanghai, China). Gene-specific primers provided in Table S9 (see online supplementary material) were used for qRT–PCR in 96-well plates (iQ5 Real Time PCR System; Bio-Rad), as described previously. The *ETF* and β-*ACTIN* genes were used as the internal reference to calculate relative gene expression [27, 55]. All analyses were performed in three biological replicates with three technical replications.

### Data analysis

The experiments were performed for at least three biological repeats. Statistical analysis was performed using either Student’s two-tailed *t*-test when comparing treatments with controls or multiple comparisons together using SPSS 19 software (IBM, Chicago, IL, USA) via one-way analysis of variance (ANOVA) at the 0.05 probability level.

## Acknowledgements

The authors acknowledge support from the National Key Research and Development Program of China (2018YFD1000601), and funding from Hunan Agricultural University.

## Authors’ contributions

J.Z. planned and designed the research, P.L., H.Z., J.M.L., J.L., M.Z., and P.H.L. performed experiments, P.L. and H.Z. analysed data. J.Z., P.L., Z.L., Q.T. and K.W. wrote and revised the article.

## Data availability

Most data that support the findings of this study are available in the supplementary material of this article, others will be available upon request from the corresponding author, who will also be responsible for the distribution of materials integral to the findings presented in this article in accordance with the policy described in the Instructions for Authors.

## Conflict of interest

None declared.

## Supplementary data

Supplementary data is available at *Horticulture Research* online.

## Supplementary Material

Web_Material_uhac278
