# Supplementary material for: Variations of stomata development in tea plant (Camellia sinensis) leaves in different light and temperature environments and genetic backgrounds
Source: Hortic Res. 2022 Dec 9;10(2):uhac278. doi: 10.1093/hr/uhac278 (PMC9926154; doi:10.1093/hr/uhac278)
Supplement: Web_Material_uhac278 [file web_material_uhac278.zip › Supplement stomata development R1.pdf]

Supplemental Fig S1:

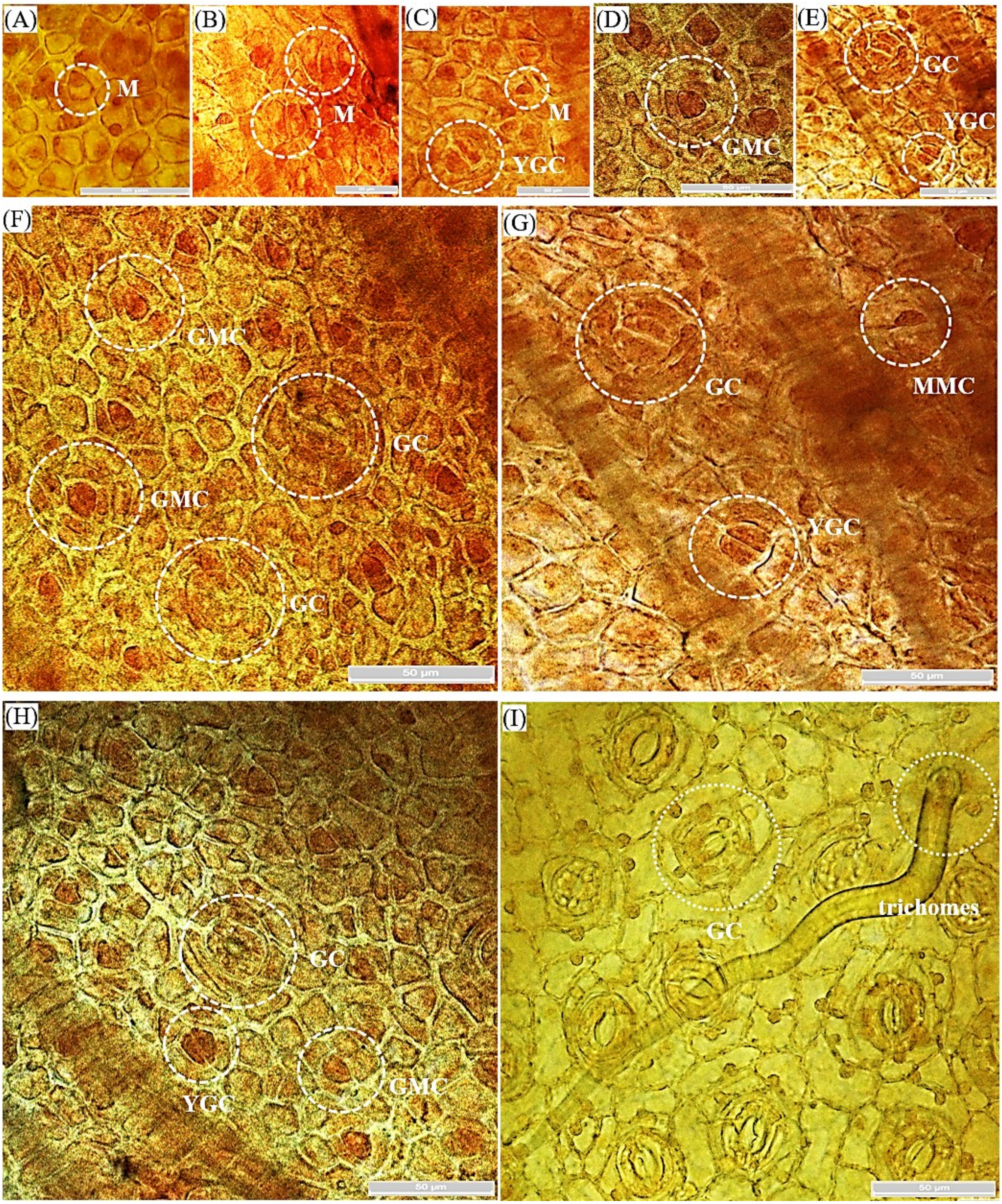

Supplemental Fig S1. stomatal formation and patterning in brightfield microscopically.

(A-I) To obtain differential interference contrast (DIC) images, tea leaves were treated with destaining solution (containing 75% ethanol and 25% acetic acid) for 8h was cleared. After a treatment the samples were via ethanol 75% for 1 h. Scale bar = 50 µm.

Supplemental Figure S2:

(A)

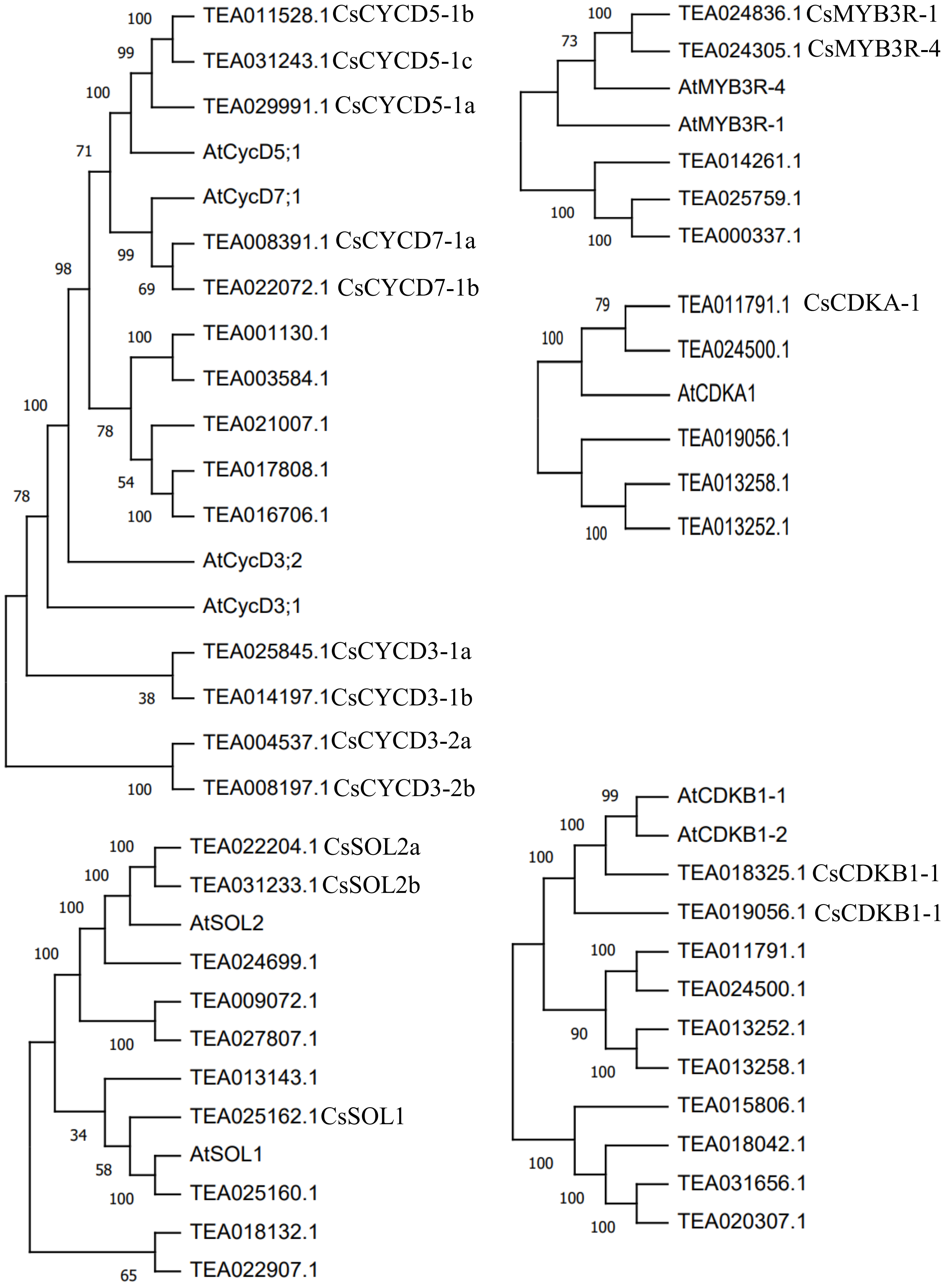

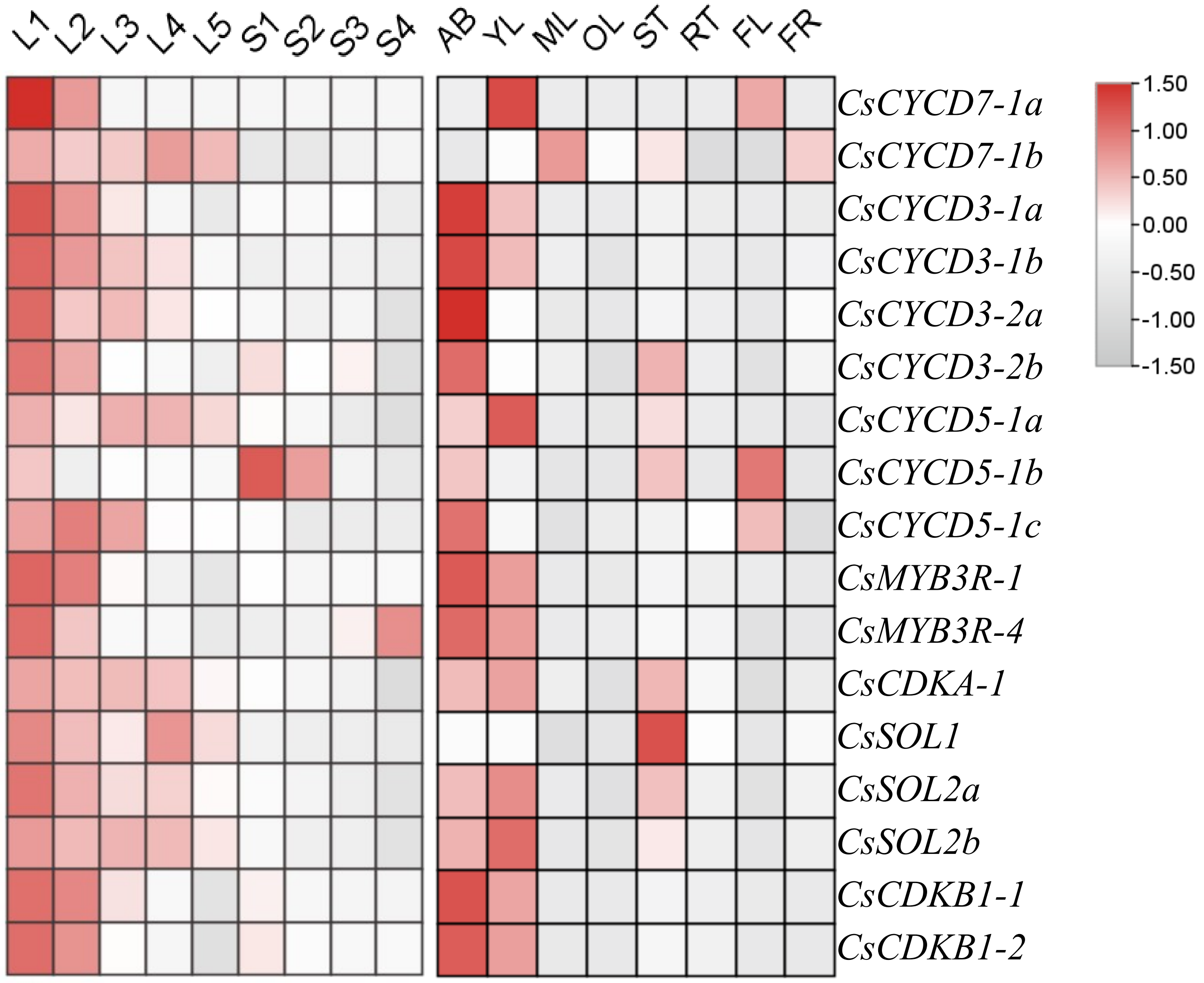

L1
L2
L3
L4
L5
S1
S2
S3
S4
AB
YL
ML
OL
ST
RT
FL
FR
CsCYCD7-1a
CsCYCD7-1b
CsCYCD3-1a
CsCYCD3-1b
CsCYCD3-2a
CsCYCD3-2b
CsCYCD5-1a
CsCYCD5-1b
CsCYCD5-1c
CsMYB3R-1
CsMYB3R-4
CsCDKA-1
CsSOL1
CsSOL2a
CsSOL2b
CsCDKB1-1
CsCDKB1-2
1.50
1.00
0.50
0.00
-0.50
-1.00
-1.50

Supplemental Figure S2:

(B)

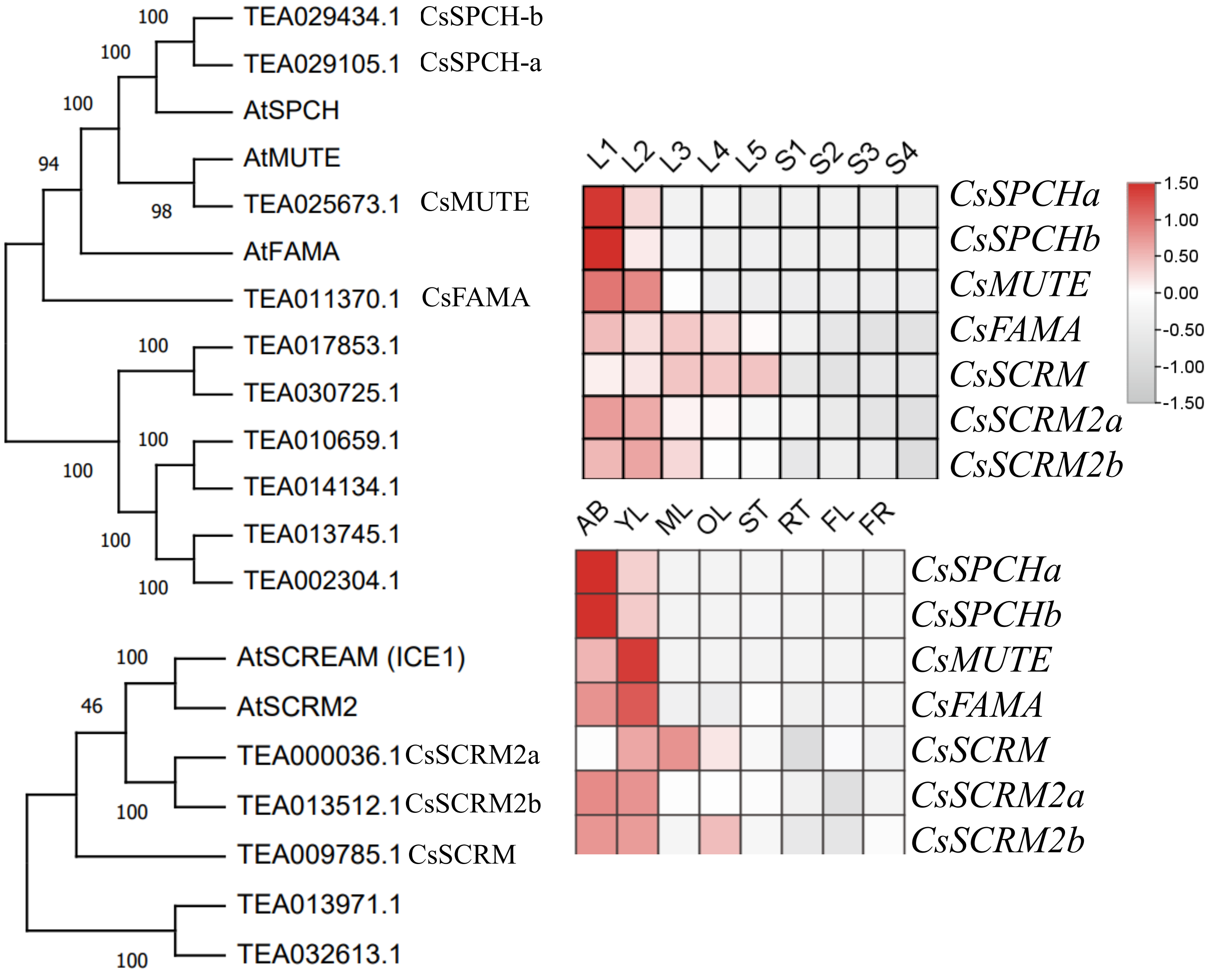

Supplemental Figure S2:

(C)

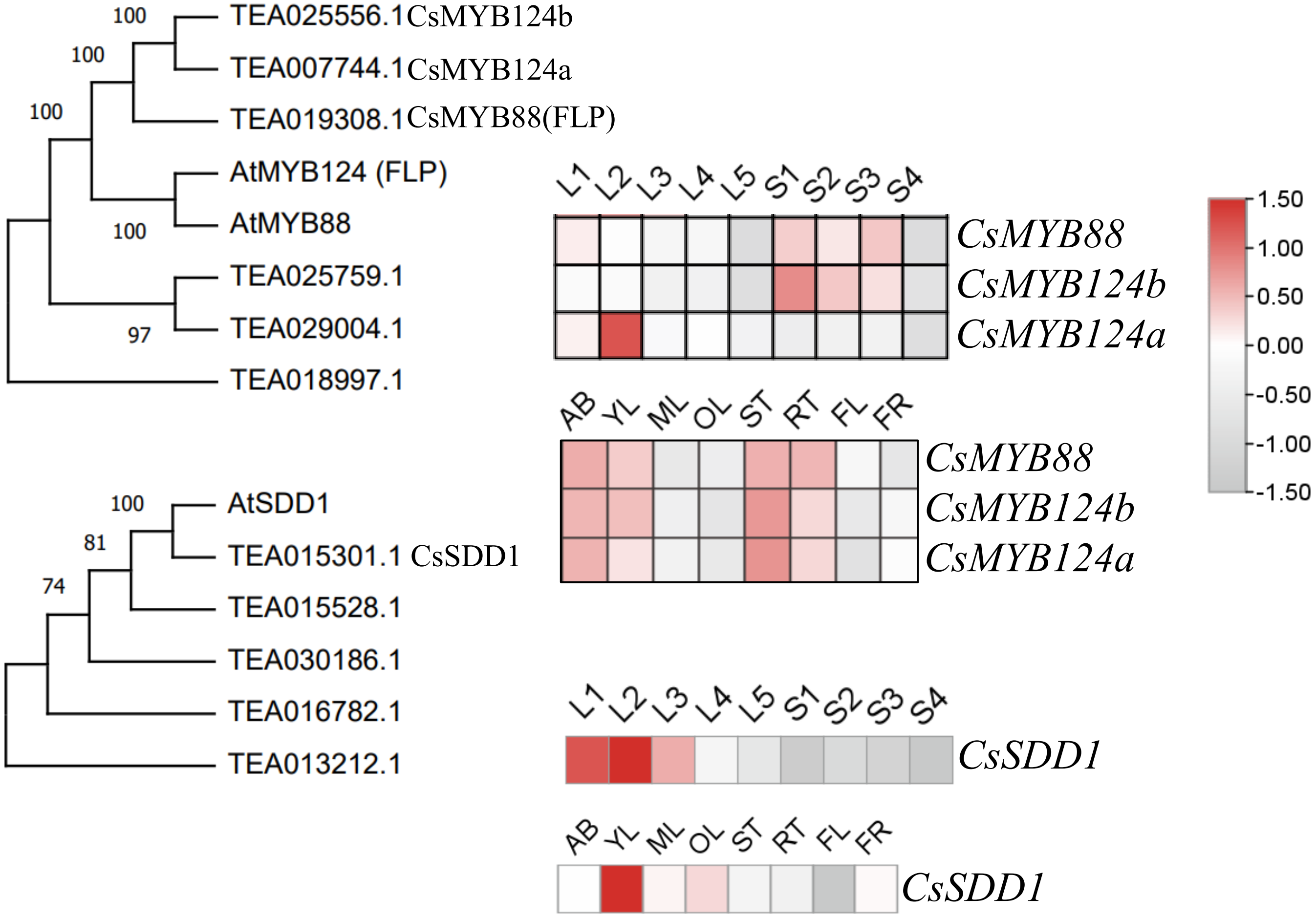

Supplemental Figure S2:

(D)

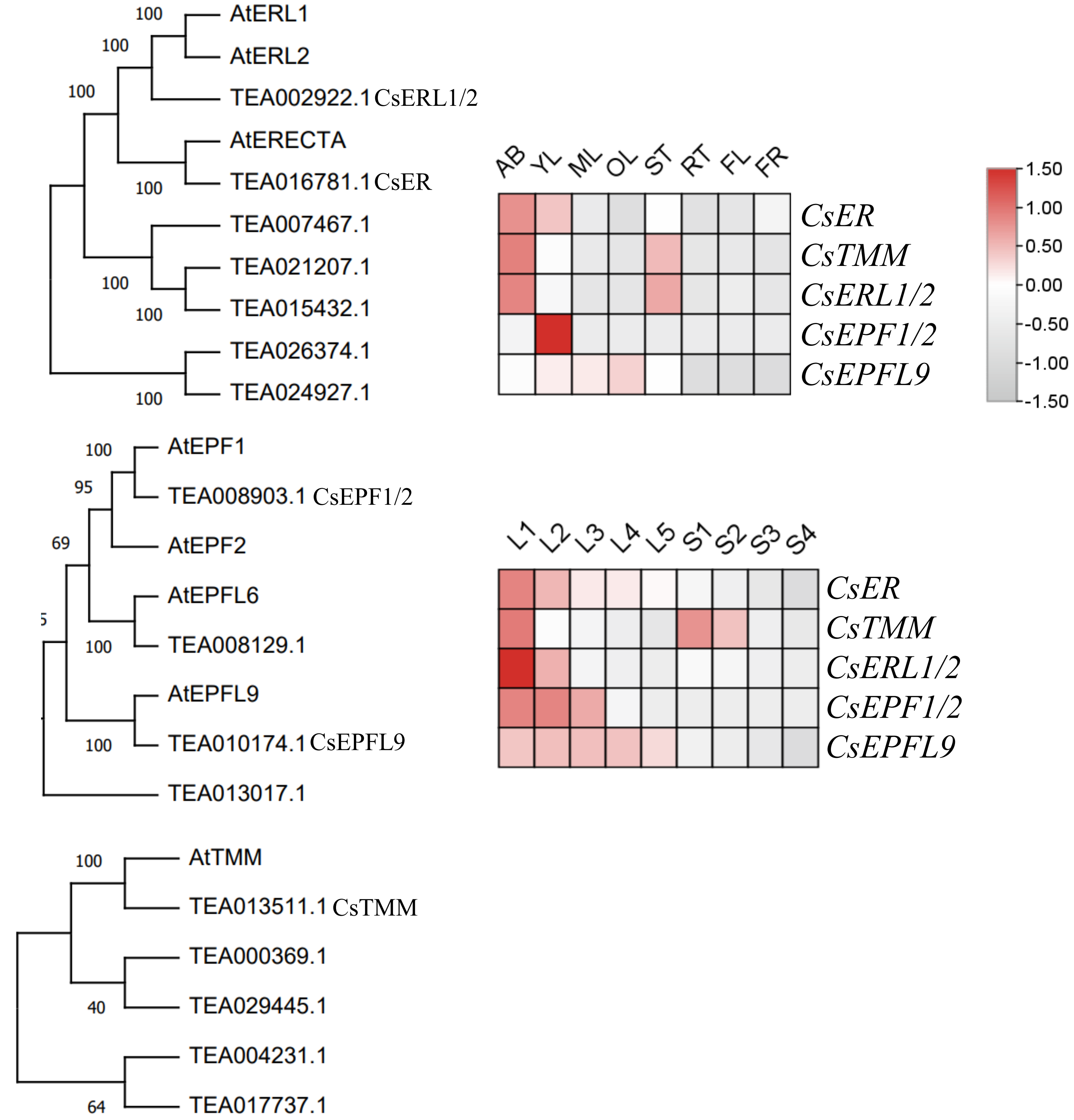

Supplemental Figure S2:

(E)

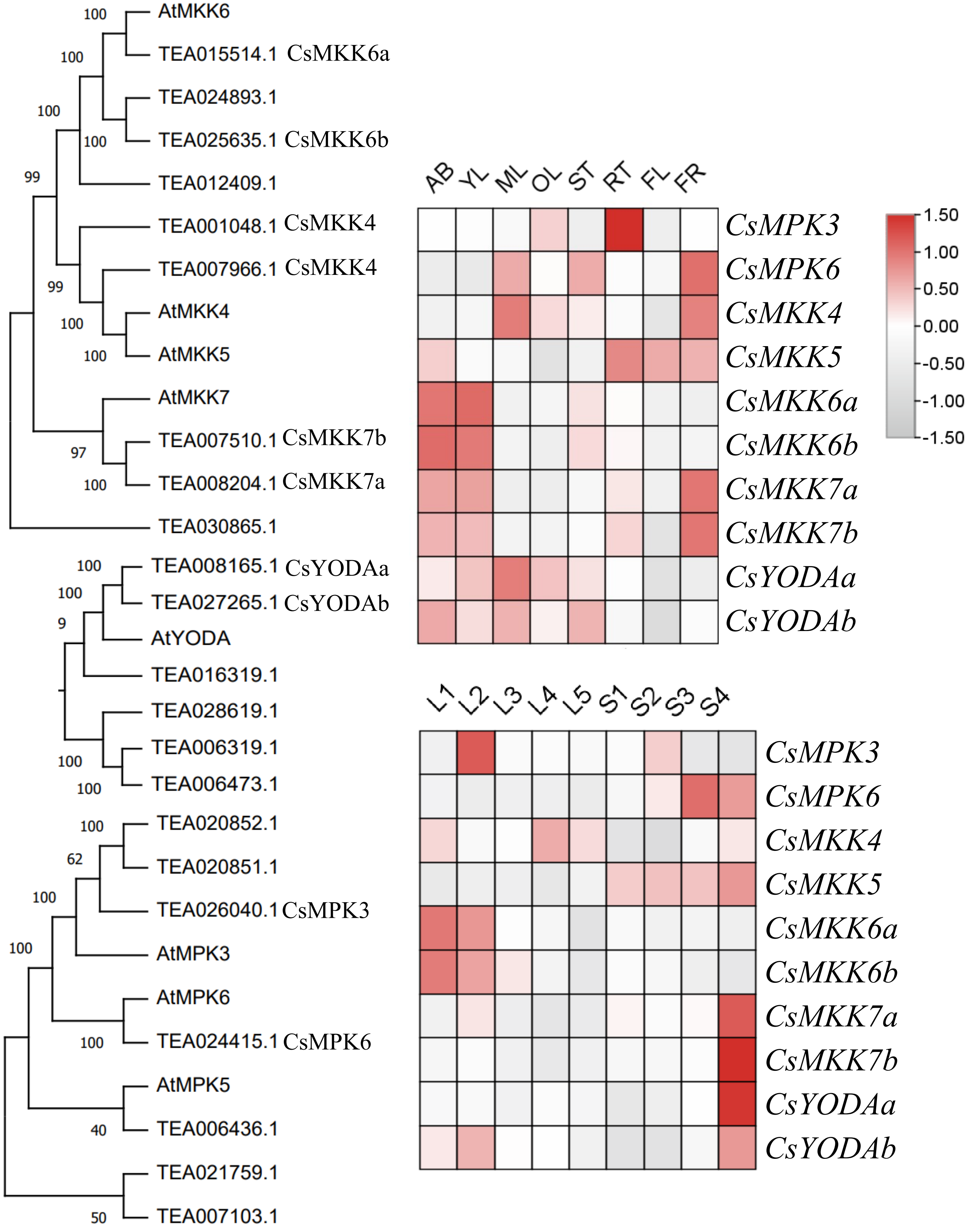

Supplemental Figure S2:

(F)

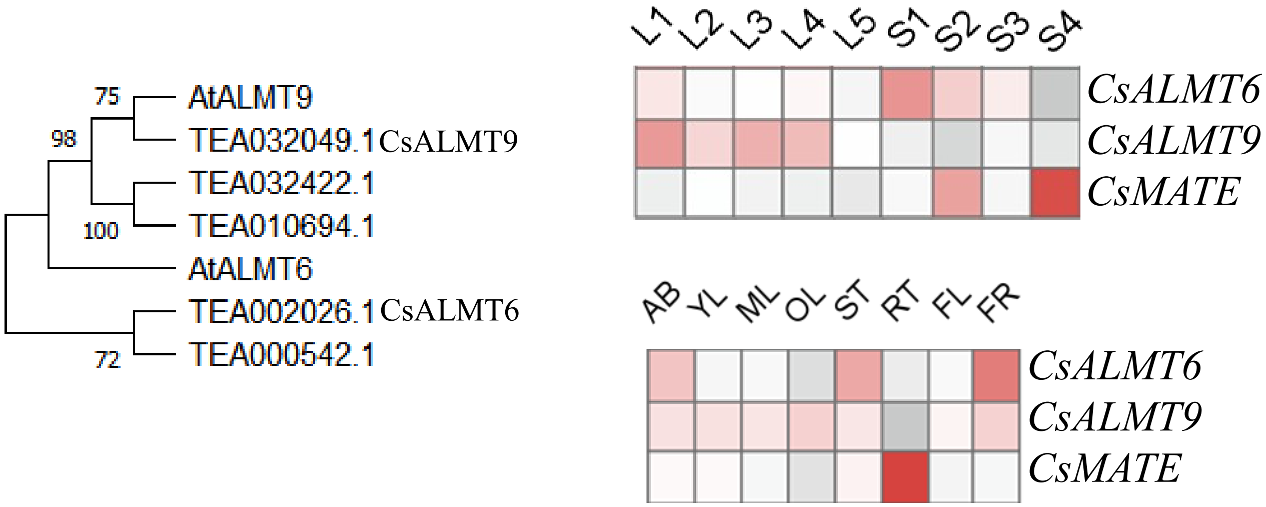

Supplemental Figure S2:

(G)

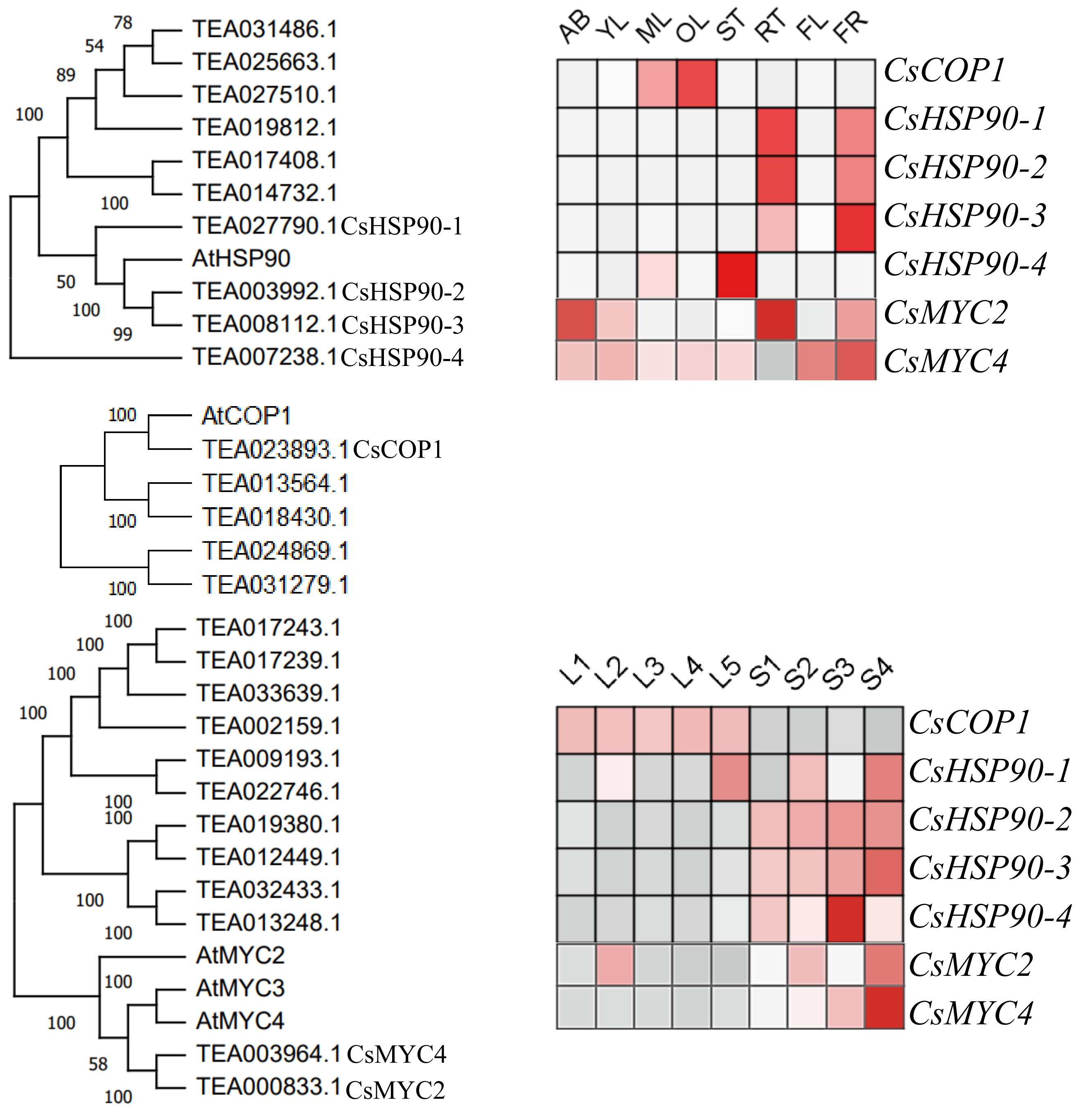

Supplemental Figure S2. The phylogenetic analysis and expression patterns of genes involved in Camellia sinensis stomatal development.

(A) cell cycle and cell division-related proteins involved in stomatal development in tea leaves.
(B) bHLH transcription factor in stomatal development in tea leaves.
(C) MYB transcription factor and SDD1 involved in stomatal development in tea leaves
(D) CsERL1/2, CsER, CsTMM, CsEPFL9 CsEPF1/2 involved in stomatal development in tea leaves.
(E) Proteins kinase involved in stomatal development in tea leaves.
(F) Involved in stomatal movement in tea leaves.
(G) CsHSP90, CsCOP1 and CsMYC2, CsMYC4 involved in stomatal development in tea leaves.
The functionally characterized Arabidopsis mays homologue proteins were used as references. Amino acid sequences were aligned by using Clustal W, and MEGA 6.0 software was used to construct the phylogenetic tree by the NJ method with 1000 bootstrap replicates. Expression of genes in different tissues of Camellia sinensis plants were retrieved from.

Supplemental Figure S3:

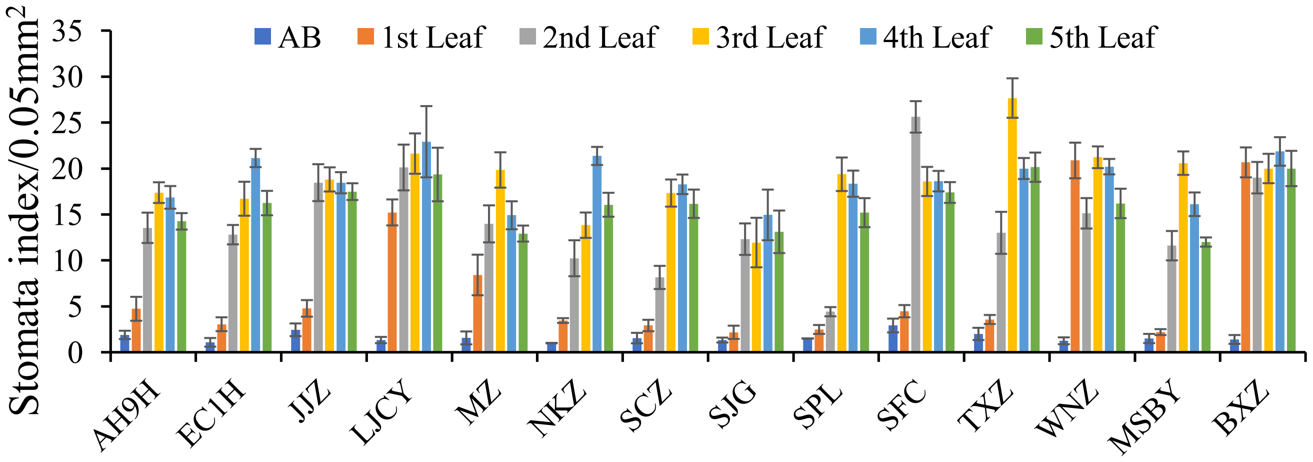

Supplemental Figure S3. Comparison of stomatal density in developing leaves, the apical buds (ABs), the 1st, 2nd, 3rd, 4th and 5th leaf of various tea plant varieties.

The stomatal density was expressed as stomata index/0.05mm^2.The tea plant varieties are: AH9, Anhui #9; ER1, Echa #1; JJZ, Jingjizong; LJCY, Longjingchangye; MZ, Meizhan; NKZ, Nongkangzao; SCZ, Shuchazao; SJG, Shuijingui; SPL, Shanpolv; SFC, Shifocui; TXZ, Texiangzao; WNZ, Wuniuzao; MSBH, Mingshanbaihao; BXZ, Bixiangzao.

Supplemental Figure S4:

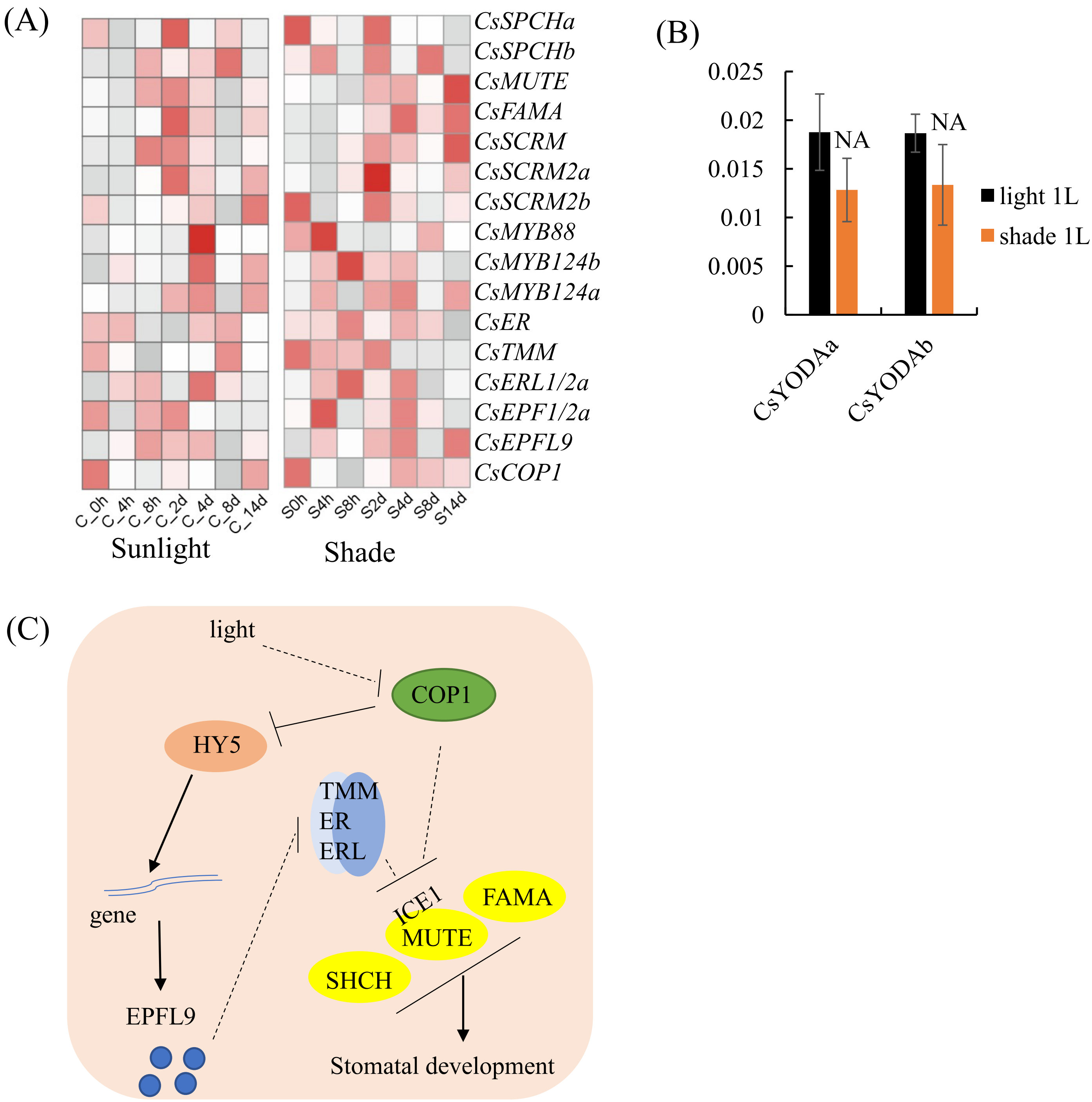

Supplemental Figure S4. Light involved in stomatal development in tea leaves.

(A) Heatmap analyses of stomatal lineage gene expression under regular sunlight and shading treatment.
(B) Expression of *CsYODAa*, *CsYODAb* genes in the 1st under the sunlight or shading treatment.
(C) Model of the light module in promoting stomatal development.

Supplemental Figure S5:

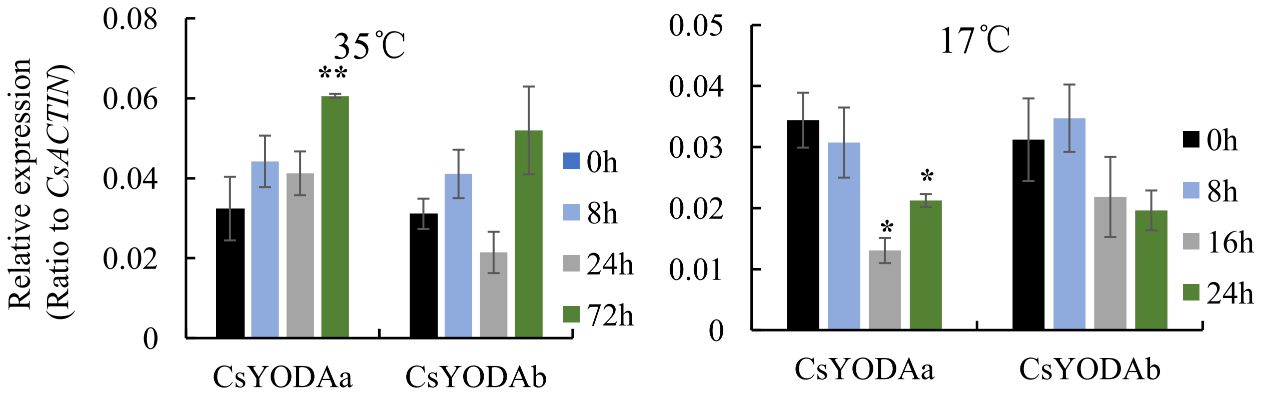

Supplemental Figure S5. Temperature involved CsYODA gene response in tea leaves.

Expression level of CsYODA in 35℃ and 17℃ treatment in tea plant. Asterisks indicate statistical significance based on Student's *t*-test; ** $P < 0.01$, * $P < 0.05$

Supplemental Figure S6:

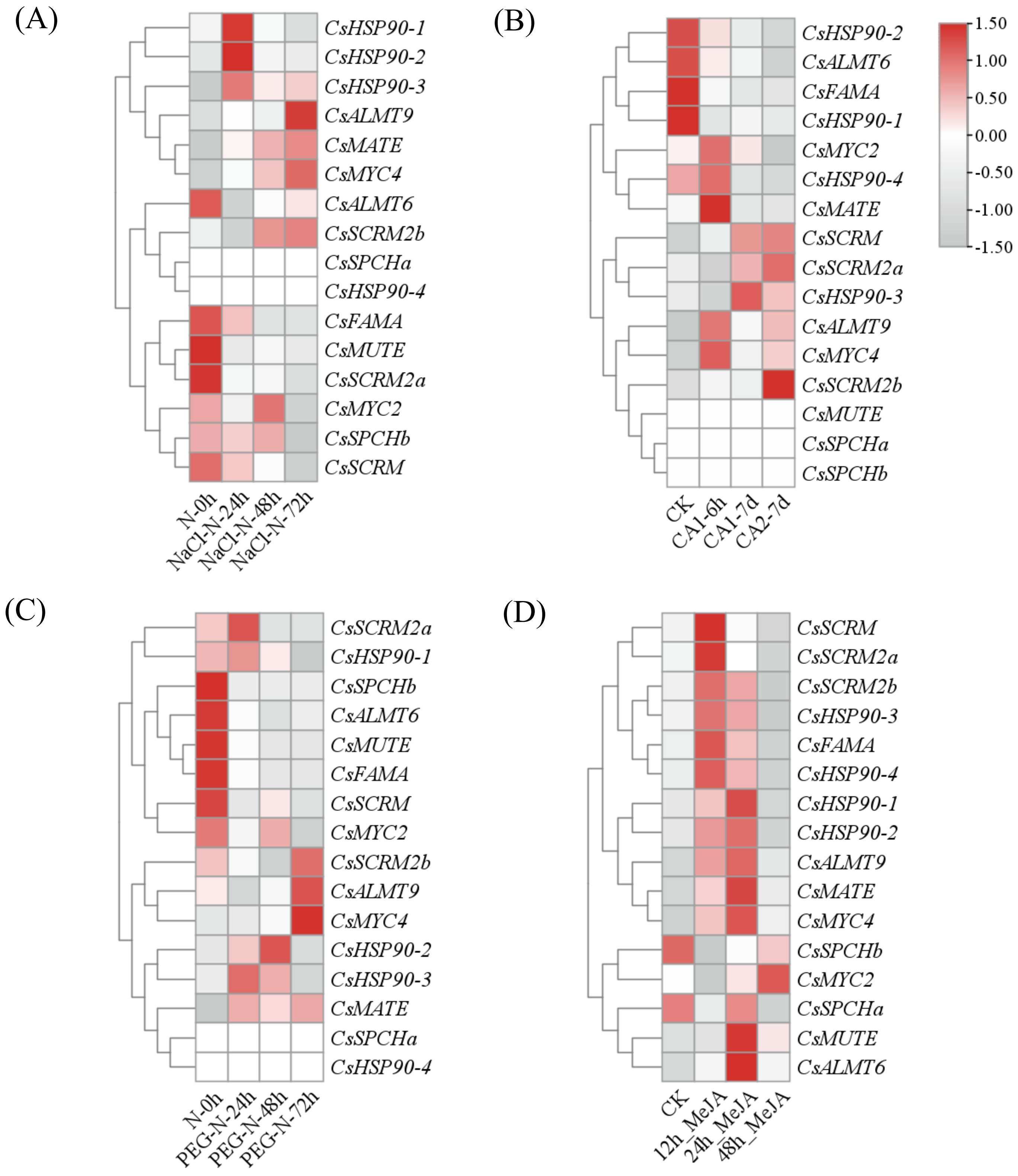

Supplemental Fig S6. Heatmap of stomatal closure gene expression under different abiotic stress treatments.

Stress treatments include methyl jasmonate (MeJA)(A), drought (PEG)(B), chilling(C) and salinity(D) stress.
The FPKM values of the Illumina RNA-seq data were reanalyzed and log2 transformed. The color scale represents the gene expression level; grey represents down-regulation, and red represents up-regulation.

Supplemental Figure S7:

(A)

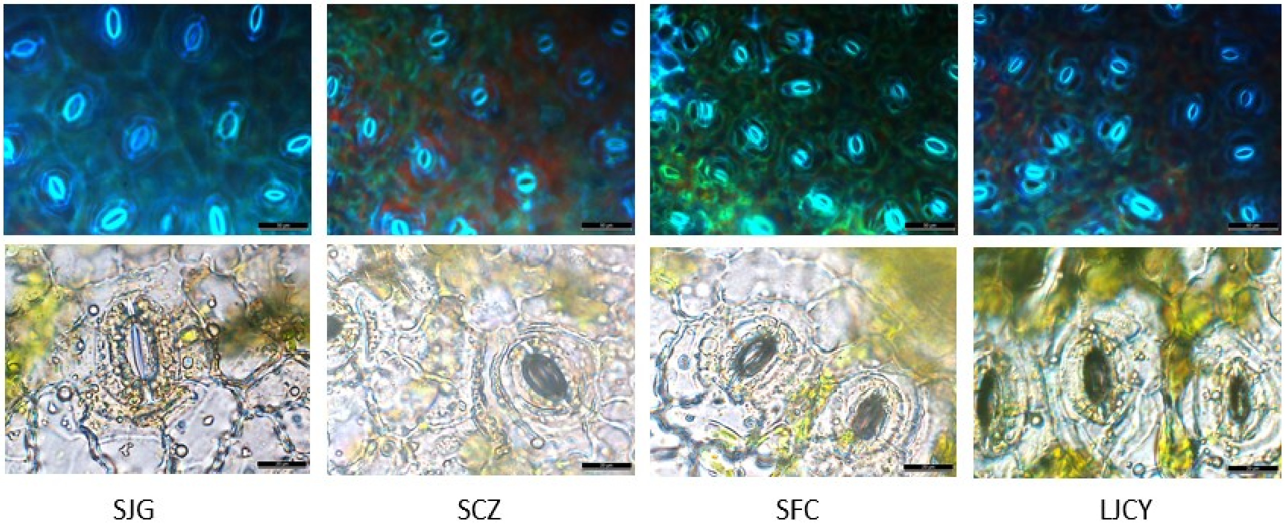

(B)

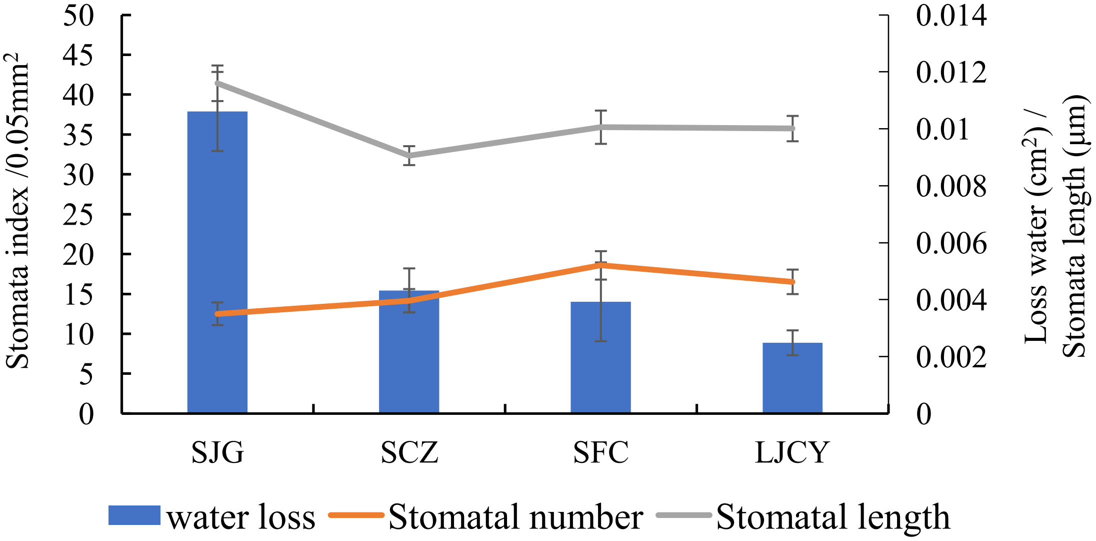

(C)

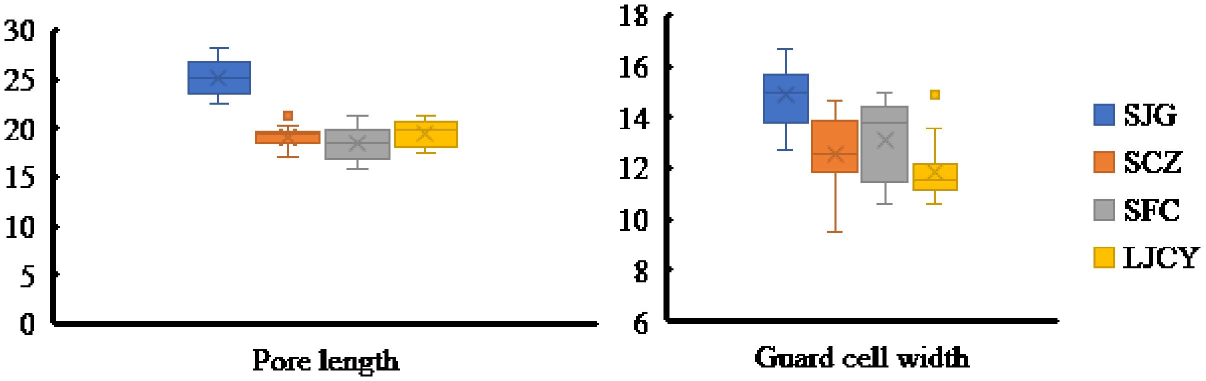

Supplemental Figure S7. Effects of stomatal size and stomatal density on leaf water loss

(A) Comparison of stomatal size to in four tea plant variety. Scale bar, 50 µm and 20 µm.
(B) Correlation of water content with stomatal density and stomatal size in leaves of four tea species.
(C) Comparison of the guard cell width and stomatal length in species with four tea species（Scale bar, µm）.

Supplemental Figure S8:

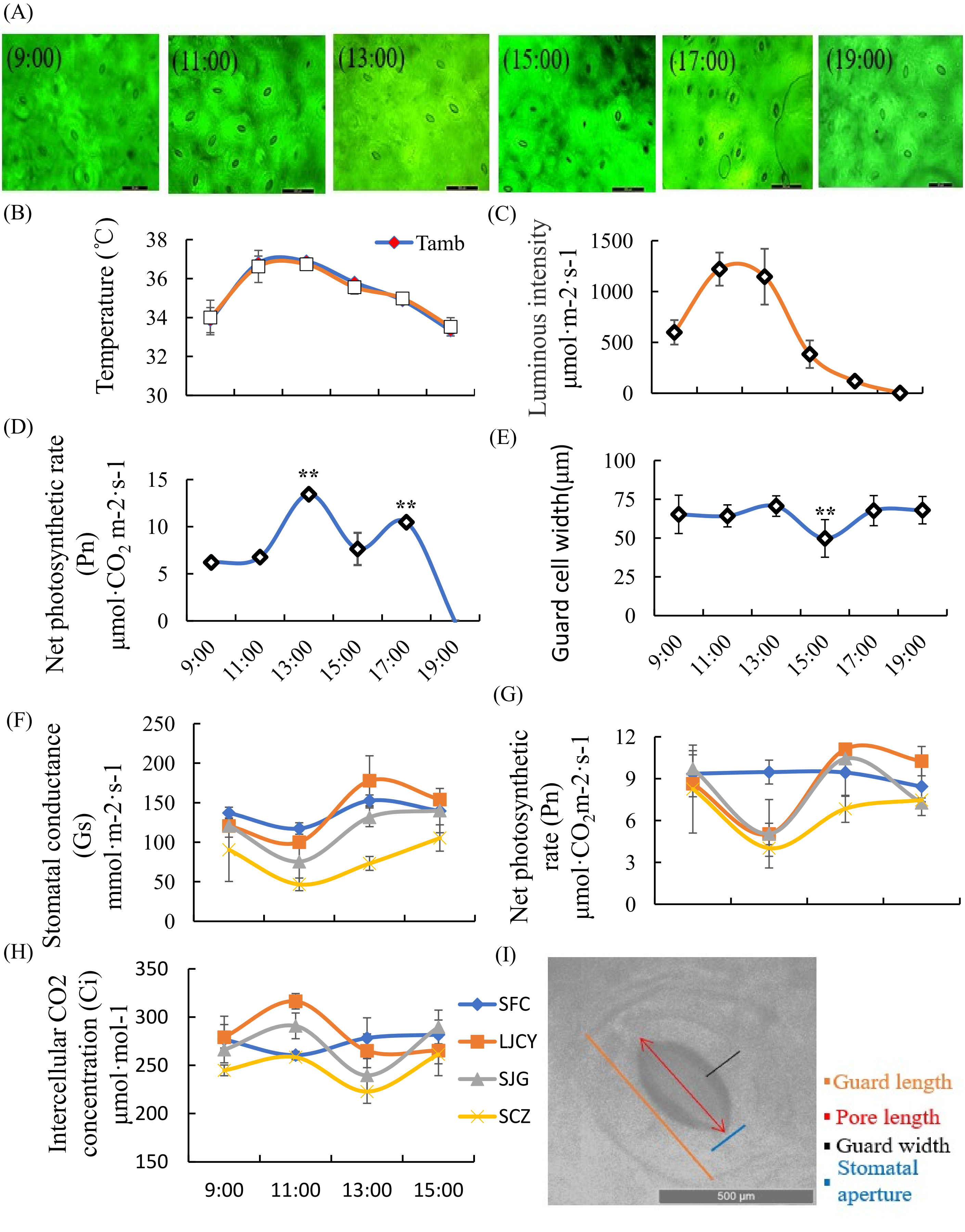

Supplemental Figure S8: Stomata movement state and leaf photosynthesis rate in tea plant varieties during the day time.
(A) The state of stomatal movement during the day in the 3 rd tea leaf. Scale bar, 50 µm.
(B) Changes in atmospheric temperature.
(C) Changes in luminous intensity.
(D) Changes of net photosynthetic rate.
(E) Changes in guard cell width.
(F) Variations in stomatal conductance of tea plant varieties
(G) Variations in net photosynthetic rate of tea plant varieties
(H) Variations in intercellular CO_2 concentration in tea plant varieties.
(I) Schematic of the stomatal parameters in tea plant. Scale bar, 500 µm.

Asterisks indicate statistical significance based on Student's *t*-test; ** $P < 0.01$, * $P < 0.05$

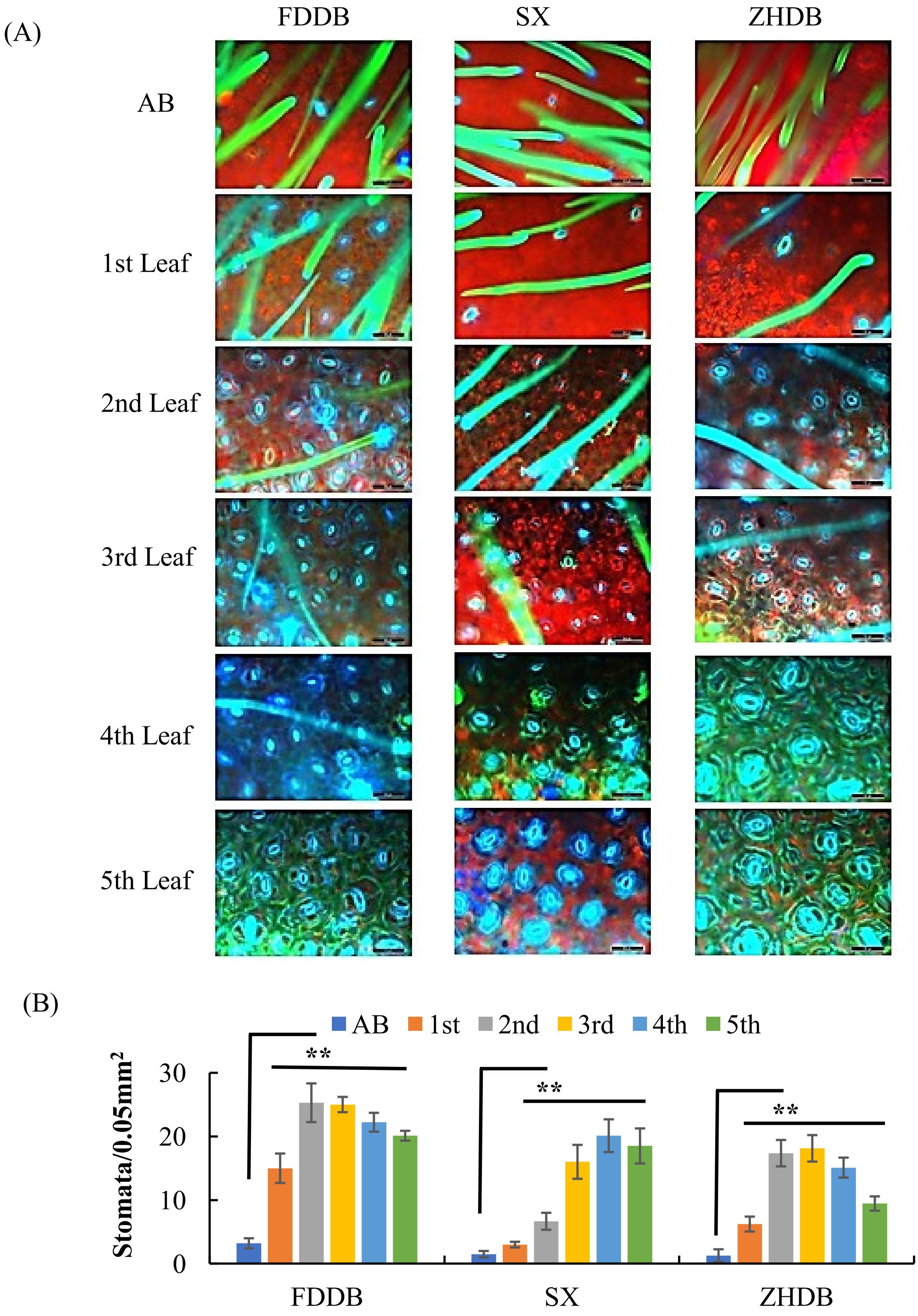
(A)
FDDB
SX
ZHDB
AB
1st Leaf
2nd Leaf
3rd Leaf
4th Leaf
5th Leaf
(B)
AB
1st
2nd
3rd
4th
5th
**
Stomata/0.05mm2
30
20
10
0
FDDB
SX
ZHDB

Supplemental Figure S9. Comparison of stomatal phenotypes in leaves of triploid tea plant varieties ZHDB and SX and diploid tea plant FDDB.

A. The images of stomata and trichomes in the apical buds (ABs), the 1st, 2nd, 3rd, 4th and 5th leaf of FDDB,SX and ZHDB. Scale bar = 50 μm.
B. Comparison of stomatal densities in developing leaves (the apical buds (ABs), the 1st, 2nd, 3rd, 4th and 5th leaf of various tea plant varieties:FDDB, fudingdabai; SX, shuixian; ZHDB, zhenghedabai. The stomatal density was expressed as stomata/0.05 mm^2.

Asterisks indicate statistical significance based on Student's *t*-test; ** $P < 0.01$, * $P < 0.05$
